# A genomic view of trophic and metabolic diversity in clade-specific *Lamellodysidea* sponge microbiomes

**DOI:** 10.1186/s40168-020-00877-y

**Published:** 2020-06-23

**Authors:** Sheila Podell, Jessica M. Blanton, Aaron Oliver, Michelle A. Schorn, Vinayak Agarwal, Jason S. Biggs, Bradley S. Moore, Eric E. Allen

**Affiliations:** 1 Marine Biology Research Division, Scripps Institution of Oceanography, University of California, San Diego, La Jolla, CA USA; 2 Laboratory of Microbiology, Wageningen University, Wageningen, The Netherlands; 3 School of Chemistry and Biochemistry and School of Biological Sciences, Georgia Institute of Technology, Atlanta, GA USA; 4 University of Guam Marine Laboratory, UoG Station, Mangilao, GU USA; 5 Center for Marine Biotechnology and Biomedicine, Scripps Institution of Oceanography, University of California, San Diego, La Jolla, CA USA; 6 Skaggs School of Pharmacy and Pharmaceutical Sciences, University of California, San Diego, La Jolla, CA USA; 7 Center for Microbiome Innovation, University of California, San Diego, La Jolla, CA USA; 8 Division of Biological Sciences, University of California, San Diego, La Jolla, CA USA

**Keywords:** *Lamellodysidea*, Sponge microbiome, Cyanosponge, *Hormoscilla*, *PBDE*, *Methylospongia*, *Prochloron*

## Abstract

**Background:**

Marine sponges and their microbiomes contribute significantly to carbon and nutrient cycling in global reefs, processing and remineralizing dissolved and particulate organic matter. *Lamellodysidea herbacea* sponges obtain additional energy from abundant photosynthetic *Hormoscilla* cyanobacterial symbionts, which also produce polybrominated diphenyl ethers (PBDEs) chemically similar to anthropogenic pollutants of environmental concern. Potential contributions of non-*Hormoscilla* bacteria to *Lamellodysidea* microbiome metabolism and the synthesis and degradation of additional secondary metabolites are currently unknown.

**Results:**

This study has determined relative abundance, taxonomic novelty, metabolic capacities, and secondary metabolite potential in 21 previously uncharacterized, uncultured *Lamellodysidea*-associated microbial populations by reconstructing near-complete metagenome-assembled genomes (MAGs) to complement 16S rRNA gene amplicon studies. Microbial community compositions aligned with sponge host subgroup phylogeny in 16 samples from four host clades collected from multiple sites in Guam over a 3-year period, including representatives of Alphaproteobacteria, Gammaproteobacteria, Oligoflexia, and Bacteroidetes as well as Cyanobacteria (*Hormoscilla*). Unexpectedly, microbiomes from one host clade also included Cyanobacteria from the prolific secondary metabolite-producer genus *Prochloron*, a common tunicate symbiont.

Two novel Alphaproteobacteria MAGs encoded pathways diagnostic for methylotrophic metabolism as well as type III secretion systems, and have been provisionally assigned to a new order, designated *Candidatus* Methylospongiales. MAGs from other taxonomic groups encoded light-driven energy production pathways using not only chlorophyll, but also bacteriochlorophyll and proteorhodopsin. Diverse heterotrophic capabilities favoring aerobic versus anaerobic conditions included pathways for degrading chitin, eukaryotic extracellular matrix polymers, phosphonates, dimethylsulfoniopropionate, trimethylamine, and benzoate. Genetic evidence identified an aerobic catabolic pathway for halogenated aromatics that may enable endogenous PBDEs to be used as a carbon and energy source.

**Conclusions:**

The reconstruction of high-quality MAGs from all microbial taxa comprising greater than 0.1% of the sponge microbiome enabled species-specific assignment of unique metabolic features that could not have been predicted from taxonomic data alone. This information will promote more representative models of marine invertebrate microbiome contributions to host bioenergetics, the identification of potential new sponge parasites and pathogens based on conserved metabolic and physiological markers, and a better understanding of biosynthetic and degradative pathways for secondary metabolites and halogenated compounds in sponge-associated microbiota.

Video Abstract

## Background

The prodigious seawater pumping capabilities of filter-feeding marine sponges contribute significantly to carbon cycling in global reef habitats, through the breakdown and remineralization of both dissolved and particulate forms of organic matter [1–3]. Nutrient recycling is facilitated by host-associated microbial communities comprised of diverse sponge-specific taxa [4], including Gamma- and Alphaproteobacteria, Acidobacteria, Actinobacteria, Chloroflexi, Cyanobacteria, Nitrospirae, Tectomicrobia, candidate phylum Poribacteria, and archaeal Thaumarchaeota (reviewed in [5, 6]).

Some sponge taxa contain abundant cyanobacterial symbionts, whose photosynthetic activities are essential for host health and growth [7, 8]. Studies measuring potential contributions of photosynthetic symbionts to host energy metabolism have historically used *Lamellodysidea herbacea* as a prototypical model [8–10]. These studies have suggested that phototrophic cyanobacteria can contribute as much as 80% of the total sponge carbon budget, although significant variation has been observed among individual sponges [11].

The cyanobacterial symbionts of *L. herbacea* are dominated by a single taxonomic clade known as *Hormoscilla spongeliae* (historically described under the genus names *Oscillatoria* and *Phormidium*), found in filamentous bundles called trichomes that can comprise up to 50% of sponge weight [8, 12]. Although *Hormoscilla* sponge symbionts have never been successfully cultured in the laboratory, two genomes of this bacterial clade have recently been assembled from trichome-enriched metagenomic samples [13]. 16S rRNA gene amplification studies have detected sponge-specific Alpha- and Gammaproteobacteria sequences associated with *Lamellodysidea* [14], but did not investigate how these non-*Hormoscilla* symbionts might contribute to metabolic activities and ecological interactions in the sponge microbiome.

*Hormoscilla* sponge symbionts have attracted particular attention for their ability to produce high concentrations of polybrominated diphenyl ethers (PBDEs), which are chemically similar to anthropogenic pollutants of environmental concern [15–19]. Laboratory studies have suggested that naturally occurring PBDEs might act as antimicrobial agents [20, 21] or as predator feeding deterrents [22], but the biologically relevant activities of these compounds in native marine habitats remain controversial.

PBDE product diversity among *Lamellodysidea* sponges is strongly correlated with the taxonomy of both sponge hosts and their *Hormoscilla* symbionts, suggesting potential co-evolutionary processes linking host genotype with microbiome and metabolome diversity [16]. The discovery that not all sponge-associated *Hormoscilla* strains produce PBDEs [15, 16] offers an opportunity to compare microbial community compositions and metabolic capabilities in PBDE-producing versus non-producing sponge hosts. The current study addresses these questions through metagenomic reconstruction of 21 new sponge-associated microbial metagenome-assembled genomes (MAGs) from *Hormoscilla*-dominated *Lamellodysidea* sponge samples, enabling detailed exploration of their potential contributions to the overall microbial community.

## Results

### Sponge samples and metagenomic assemblies

**Fig. 1:**
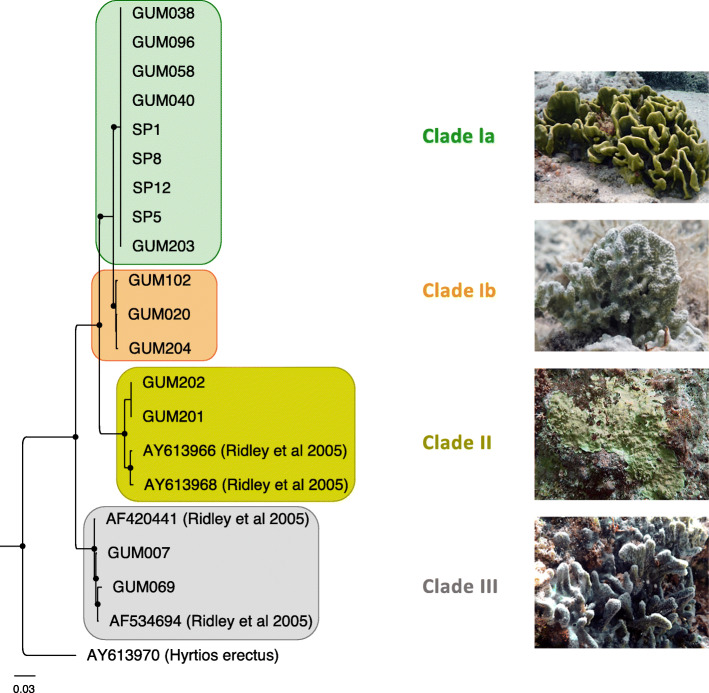
Phylogenetic tree of *Lamellodysidea* sponge samples. The tree is based on gene amplification of ITS-2 gene sequences, with *Hyrtios erectus* as an outgroup. Black dots indicate nodes with bootstrap values exceeding 0.8. Representative samples shown in photographs: clade Ia, SP5; clade Ib, GUM020; clade II, GUM201; clade III, GUM007

*L. herbacea* sponge samples were collected over a 3-year period from four different coastal locations in Guam, including both summer and winter seasons (Supplementary Table S1, Additional file 1). Based on a phylogenetic tree of internal transcribed spacer (ITS) regions (Fig. 1), these samples included representatives of all four previously described *L*. *herbacea* clades [16]. Clade Ia and Ib samples, whose microbial communities have not previously been described, were obtained at multiple different times and locations. Samples from clades II and III, previously analyzed by 16S rRNA gene amplification [14], are represented by tissue samples taken from different individual sponges collected on the same dates at a single site.

**Fig. 2:**
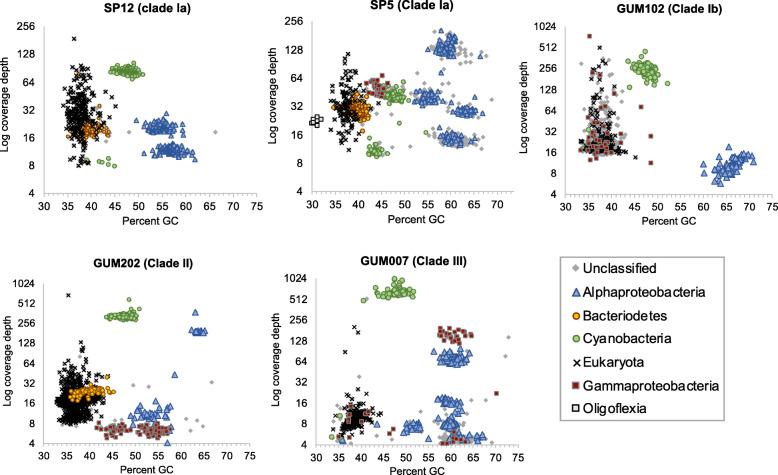
Phylum/class-level taxonomic abundance in assembled holobiont metagenomes. Binning includes all scaffolds > 10,000 nt. Higher coverage values (*y*-axis) indicate greater relative abundance. Phylum- and class-level taxonomic assignments were made based on DarkHorse software analysis of predicted protein matches to previously classified sequences from the GenBank nr database, accessed January 2, 2019

Assemblies for each metagenomic sample were initially performed using all available reads for that sample (Supplementary Table S2, Additional file 1). Combined properties of read coverage, nucleotide composition, and predicted protein matches to the GenBank nr database revealed a relatively small number of discrete scaffold clusters, mapping primarily to database sequences from Cyanobacteria, Bacteroidetes, Alphaproteobacteria, Gammaproteobacteria, and Oligoflexia (Fig. 2). Scaffolds derived from host sponge DNA were present at similar coverage depths to bacterial clusters, but were distinguishable by nucleotide composition and predicted matches to reference database sequences.

Metagenomic scaffold bins were used to recruit matching raw read pairs that were subsequently used in targeted subassemblies to produce 23 consensus population genomes (Table 1; Supplementary Table S3, Additional file 1). All of the assembled genomes except one (clade Ib: GM102ARS1) met or exceeded MIMAG standards for high- or medium-quality drafts [23]. CheckM analysis [24] estimated that 16 of the 23 MAGs were more than 90% complete, and 19 contained near full-length 16S rRNA gene sequences. The exceptional quality of these MAGs enabled detailed comparisons with both genomically sequenced relatives and environmental 16S rRNA gene surveys.

**Table 1:** Metagenome-assembled genome properties

Host clade	MAG name	Total length	Pct GC	Num scf	Gene count	16S len	CheckM complete	CheckM contam	Taxonomy
Ia	SP12AHP1	2,316,397	54.6	125	2323	1467	91.4	0.0	Alphaproteobacteria; Rhodobacterales; Hyphomonadaceae
Ia	SP12ARB1	2,913,125	57.2	262	3307	1381	69.1	0.0	Alphaproteobacteria; Rhodobacterales; Rhodobacteraceae
Ia	SP12BCY1	2,978,903	39.9	202	2868	1517	100.0	0.0	Bacteroidetes; Cytophagia; Cytophagales; Cytophagaceae; Ekhidna
Ia	SP12CHS1	4,999,041	48.0	573	6155	1486	96.6	0.2	Cyanobacteria; Oscillatoriophycideae; Oscillatoriales; Gomontiellaceae; Hormoscilla
Ia	SP5AHP2	2,126,940	54.9	153	2340	1467	87.8	0.0	Alphaproteobacteria; Rhodobacterales; Hyphomonadaceae
Ia	SP5ARB1	2,540,908	59.8	118	2682	1415	82.4	3.4	Alphaproteobacteria; Rhodobacterales; Rhodobacteraceae
Ia	SP5ARS3	2,817,712	63.5	46	2617	n.d	94.8	0.0	Alphaproteobacteria: Rhodospirillales
Ia	SP5BCY1	2,936,924	39.7	200	2813	1517	96.4	0.5	Bacteroidetes; Cytophagia; Cytophagales; Cytophagaceae; Ekhidna
Ia	SP5CHS1	4,838,078	47.7	445	5922	1484	96.6	0.2	Cyanobacteria; Oscillatoriophycideae; Oscillatoriales; Gomontiellaceae; Hormoscilla
Ia	SP5CPC	2,787,143	43.8	397	3056	1442	77.4	0.0	Cyanobacteria; Synechococcales; Prochloraceae; Prochloron
Ia	SP5GCR1	1,621,865	44.7	28	1518	1542	98.3	0.0	Gammaproteobacteria; Chromatiales
Ia	SP5OBV1	1,589,243	29.8	57	1544	1469	91.2	0.0	Oligoflexia; Bdellovibrionales
Ib	GM102ARS1	1,092,149	65.2	302	1180	263	41.2	0.8	Alphaproteobacteria: Rhodospirillales
Ib	GM102HS1	5,041,890	47.5	518	6591	227	95.7	1.8	Cyanobacteria; Oscillatoriophycideae; Oscillatoriales; Gomontiellaceae; Hormoscilla
II	GM202ARS1	2,820,038	63.5	18	2912	1500	100.0	0.0	Alphaproteobacteria: Rhodospirillales
II	GM202ARS2	1,828,912	53.3	49	1885	1496	85.3	0.8	Alphaproteobacteria: Candidatus Methylospongiales
II	GM202BCY1	3,008,284	40.3	122	2918	1517	94.9	0.5	Bacteroidetes; Cytophagia; Cytophagales; Cytophagaceae; Ekhidna
II	GM202CHS1^a^	6,869,623	47.5	70	7441	1492	97.4	2.0	Cyanobacteria; Oscillatoriophycideae; Oscillatoriales; Gomontiellaceae; Hormoscilla
III	GM7ARB1	2,553,113	60.7	59	2499	1463	96.6	0.0	Alphaproteobacteria; Rhodobacterales; Rhodobacteraceae
III	GM7ARB2	2,578,993	59.9	37	2551	1235	93.7	0.9	Alphaproteobacteria; Rhodobacterales; Rhodobacteraceae
III	GM7ARS4	1,589,243	52.0	24	1425	66	91.0	0.8	Alphaproteobacteria: Candidatus Methylospongiales
III	GM7CHS1^b^	6,299,679	47.8	6	6160	1484	100.0	1.8	Cyanobacteria; Oscillatoriophycideae; Oscillatoriales; Gomontiellaceae
III	GM7GCV1	1,911,341	61.0	40	1792	1524	89.1	0.0	Gammaproteobacteria; Cellvibrionales

Additional metadata and genome quality information are provided in Supplementary Table 3; *n.d.* not detected. Symbols denote genomes previously described in [13]

^a^MAG GUM202CHS1 is identical to NCBI accession RFFC00000000.1

^b^MAG GM7CHS1 was newly assembled as described in the “Methods” section, replacing NCBI accession RFFB00000000.1

### Taxonomic classification of assembled genomes

**Fig. 3:**
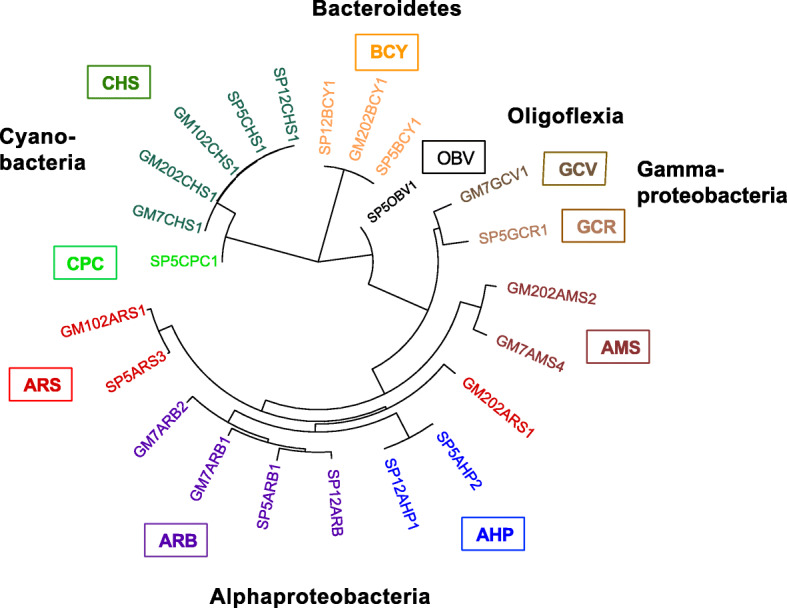
Concatenated multi-locus PhyloPhlAn tree of assembled MAG taxonomic diversity. Assembled genome group name abbreviations, in clockwise order: BCY, Bacteroidetes Cytophagaceae; OBV, Oligoflexia Bdellovibrionales; GCV, Gammaproteobacteria Cellvibrionales; GCR, Gammaproteobacteria Chromatiales; AMS, Alphaproteobacteria Methylospongia; AHP, Alphaproteobacteria Hyphomonadaceae; ARB, Alphaproteobacteria Rhodobacteraceae; ARS, Alphaproteobacteria Rhodospirillales; CPC, Cyanobacteria *Prochloron*; CHS, Cyanobacteria *Hormoscilla*. More detailed trees for each phylum-level group are provided in Supplementary Figures 1-5

Taxonomic classifications for assembled MAGs were established using concatenated multi-locus phylogenetic trees of conserved proteins and matrices of 16S rRNA gene and average amino acid identity (AAI) scores (Fig. 3; Supplementary Figures S1-5, Additional file 1). AAI scores were especially valuable in providing taxonomic classification levels for genomes with missing or incomplete 16S rRNA genes and those with few sequenced relatives, according to quantitative threshold ranges previously established for large numbers of database examples [25, 26].

**Fig. 4:**
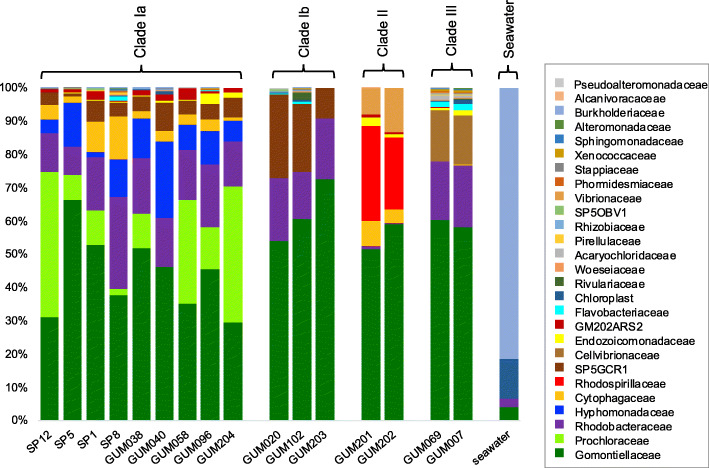
Relative abundance of family-level taxonomic groups. Based on blastn matches to 16S rRNA gene sequences in the SILVA reference database, version 132. Seawater values were obtained from sample GUM061 (Supplementary Table 1). The GM102ARS1, GM102CHS1, SP5ARS3, and GM7ARS4 MAGs did not include any sequences containing the primer-amplified 16S rRNA gene region, so these taxa are not included in this analysis. Relative abundances of these sequences, based on Bowtie2 recruitment of metagenomic reads, are shown in Supplementary Figure 7

Cyanobacteria genomes from the genus *Hormoscilla* were present in all samples, as expected, but sample SP5 from clade Ia also yielded a genome from the genus *Prochloron* (SP5CPC1; Supplementary Figure S1, Additional file 1). *Prochloron* are well-known tunicate symbionts [27], but have not previously been discussed in molecular studies of sponge microbiomes. Although *Prochloron* bacteria were first observed microbiologically on the surfaces of both marine sponges and sea cucumbers more than 30 years ago [28, 29], no sponge-derived matches to PCR-amplified (Fig. 4) or metagenomically assembled *Prochloron* 16S rRNA genes from the SP5CPC1 genome were identified at 97% or greater nucleotide identity in the GenBank nr database (Supplementary Table S4, Additional file 1) or the Sponge Microbiome Project Database [30, 31] (Supplementary Table S5, Additional file 1). The *Prochloron* 16S rRNA gene sequences obtained in this study do not contain any mismatches to commonly used amplification primers that might explain their absence from these databases, but we are also not aware of any prior 16S rRNA gene amplification studies that included *Lamellodysidea herbacea* samples from clade Ia.

Genomes SP12BCY1, SP5BCY1, and GM202BCY1 were assigned to the Bacteroidetes family Cytophagaceae (Supplementary Figure S2, Additional file 1). Based on AAI scores greater than 98%, these three genomes represent members of the same species. Their closest cultured relative was *Ekhidna lutea*, a free-living aerobic heterotroph isolated from seawater [32]. 16S rRNA gene identities of 95% and AAI scores of 67% suggest membership in the same genus as *E. lutea*.

Four Alphaproteobacteria genomes (SP12ARB1, SP5ARB1, GM7ARB1, GM7ARB2) were classified in family Rhodobacteraceae (Supplementary Figure S3, Additional file 1). Their most closely related cultured isolate was *Nioella sediminis*, a free-living aerobic marine bacterium from a sister genus of the Roseobacteria clade [33, 34]. Alphaproteobacteria genomes SP5ARS3, GM102ARS1, and GM202ARS1 fell within family Rhodospirillaceae. Terrestrial plant-associated *Azospirillum brasilense* [35] was their closest sequenced relative. Other members of genus *Azospirillum* have been identified in environmental samples from marine habitats, but not yet cultured or sequenced. An AAI score of 87% suggests that SP5ARS3 should be classified in the genus *Azospirillum*, but GM102ARS1 and GM202ARS1, with AAI scores of 49–51%, represent a new genus within the family Rhodospirillaceae. Alphaproteobacterial genomes SP12AHP2 and SP5AHP1 were classified in the Hyphomonadaceae family, as a sister genus to closest isolate relative *Hellea balneolensis*, an aerobic, heterotrophic bacterium isolated from surface seawater [36]. SP12AHP2 and SP5AHP1 have identical 16S rRNA genes and AAI scores of 99%, qualifying them as members of the same species in a new genus of family Hyphomonadaceae (Supplementary Figure 2B, Additional file 1).

GM7GCV1 was the only genome sequenced from the Gammaproteobacteria order Cellovibrionales (Supplementary Figure 4, Additional file 1). Its closest sequenced relative, *Halieaceae* LZ-16-2, is an uncharacterized bacterium obtained from a mixed laboratory culture with the saxitoxin-producing dinoflagellate *Alexandrium tamarense* (NZ_RFLW00000000.1). AAI scores of 50–65% with other members of family *Halieaceae* suggest GM7GCV1 might be classified as a new genus within this family, but 16S rRNA gene identities of 88–89% imply a more distant relationship, potentially in a new family.

The remaining community genomes were too distant from reference database examples to allow precise taxonomic assignments. GM7GCR1 had no close matches among sequenced isolates, being most closely related to unclassified environmental Gammaproteobacteria, with 16S rRNA gene nucleotide sequence identities of 90% and AAI scores below 45% (Supplementary Figure 3 and Supplementary Tables S4-5, Additional file 1). These results suggest it should be classified as either a new family within Chromatiales or a new order within Gammaproteobacteria.

Two Alphaproteobacteria genomes (GM202ARS2, GM7ARS4) fell outside any previously established orders, and could only be assigned at the class level, although their 55% AAI value when compared to each other implies they might be members of a single family (Supplementary Figure S2, Additional file 1). Genome SP5OBV1 was most closely related to *Bdellovibrio bacteriovorus* from the order Bdellovibrionales, but low 16S rRNA gene identity (82%) and AAI (40%) scores suggest it might represent a new, previously unreported order within the recently described Proteobacteria class Oligoflexia (Supplementary Figure S5, Additional file 1) [37]. Verification of these taxonomic assignments will require the discovery of additional sequences for closely related taxa.

16S rRNA genes from Rhodobacteriaceae and Rhodospirillaceae genomes were conserved in samples obtained over 3 years in the current study, and also matched previously reported sequences from a 2005 amplification study of Guam *Lamellodysidea* sponges [14] at 98–100% identity (Supplementary Table S4, Additional file 1). No 16S rRNA gene matches to the Cytophagaceae, Cellvibrionales, Hyphomonadaceae, and Bdellovibrionales genomes from this study were detected in the 2005 *Lamellodysidea* microbiome study, but this may be due to the absence of samples from host clades Ia and Ib and the small number of clones analyzed (< 40 per sample) in this earlier work [14]. The closest GenBank 16S rRNA gene matches to MAGs in the current study were associated with *Ircinia*, *Tethya. Stylissa*, and *Axinella* sponges, at 90–96% identity levels (Supplementary Table S4, Additional file 1). Additional low abundance 16S rRNA gene matches were present at 97% identity in numerous other sponge genera from the Sponge Microbiome Project Database (Supplementary Table S5, Additional file 1). No species-level matches were detected in this database for 16S rRNA gene sequences from GM7GVC1 or SP5CPC1, but more distant matches were found at 95% identity, suggesting the presence of bacteria from the same genera but not the same species [38].

### Relative abundance comparisons between samples

Microbial community compositions in all samples were strongly correlated with host clade taxonomy (Fig. 4). The most taxonomically diverse communities were observed in host clade Ia, which included two bacterial taxa that were absent from all other clades: alphaproteobacterial family Hyphomonadaecae and cyanobacterial genus *Prochloron*. Host clade II samples were notable for the consistent replacement of Alphaproteobacteria family Rhodobacteraceae with Rhodospirillaceae. Sponge metagenome 16S rRNA gene sequences from assembled MAGs accounted for 44–88% of all bacterial amplicon sequence variants (ASVs) in sponge samples, but only 0.1% in nearby seawater (Supplementary Figure S6, Additional file 1).

Relative abundances of the four MAGs with incomplete or missing 16S rRNA sequences (GM102ARS1, GM102CHS1, SP5ARS3, and GM7ARS4) were assessed by recruitment of unassembled metagenomic reads to assembled MAGs (Supplementary Figure S7, Additional file 1). Metagenomic read recruitment is known to underestimate relative abundance of genomes with smaller sizes, but 16S rRNA gene amplification over-reports taxa with multiple gene copies, precluding exact numerical agreement. Despite these differences, results from both procedures were consistent in showing the host clade-specificity of taxonomic compositions, including greater diversity and the unique presence of *Prochloron* and Hyphomonadaceae in clade 1a, as well as the replacement of Rhodobacteraceae with Rhodospirillaceae in clade II samples. These patterns were consistent over all available sample collection time points and locations tested.

### Predicted bacterial lifestyles

**Fig. 5:**
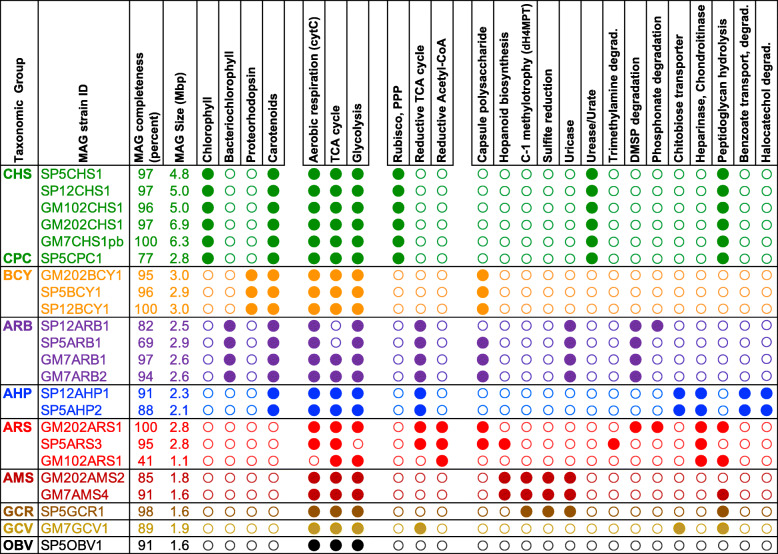
Distinctive features of *Lamellodysidea*-associated genome groups. MAG completeness was assessed using CheckM, as described in the “Methods” section. Filled/open circles indicate the presence/absence of key molecules and complete pathways. Colors indicate taxonomic groups, according to abbreviations defined in the legend to Fig. 3. Other abbreviations: cytC, cytochrome C; PPP, pentose phosphate pathway; dH4MPT, methylene-tetrahydromethanopterin dehydrogenase-dependent oxidation pathway

Predicted protein annotations for the 23 microbial genomes in this study were used to explore both broad, community-wide patterns and detailed metabolic pathways specific to individual genomes (Fig. 5). Shared features among all taxonomic groups included the presence of aerobic respiration, glycolysis, and TCA cycle enzymes. None of the assembled genomes contained genes encoding flagellar biosynthesis, although genes for gliding motility were found in all Bacteroidetes family Cytophagaceae genomes (group BCY), and twitching motility in Cyanobacteria (groups CHS and CPC), one of the Gammaproteobacteria (group GCV), and Oligoflexia (group OBV) genomes (Supplementary Table S6, Additional file 1). Several genome groups contained expanded gene families encoding adhesive molecules with the potential to resist shear forces from high seawater flow rates, for example genes encoding cadherin and ankyrin domains in all Bacteroidetes family Cytophagaceae (BCY) and Gammaproteobacteria group GCR genomes, as well as type IV pilus structures in Cyanobacteria, Alphaproteobacteria *Candidatus Methylospongiales*, Gammaproteobacteria, and Oligoflexia genomes (groups CHS, CPC, AMS, GCR, GCV, and OBV). The absence of nitrogenase complex genes in any of the assembled MAGs, combined with the near-universal presence of ammonia transporters, suggests ready availability of fixed nitrogen, consistent with detection of ammonia excretion in many sponge species [2].

Metabolic phenotype analysis of the assembled genomes identified pathways associated with phototrophic, methylotrophic, heterotrophic, and parasitic or pathogenic lifestyles. Phototrophic pathways included not only chlorophyll-based photosynthesis with carbon fixation via the Calvin-Benson cycle in *Hormoscilla* and *Prochloron* cyanobacterial genomes, but also bacteriochlorophyll-mediated anoxygenic photosynthesis in alphaproteobacterial Rhodobacteriaceae, coupled with carbon fixation via the reductive TCA cycle. A third mode of light-driven energy production was identified in Bacteroidetes family Cytophagaceae genomes, supporting ATP synthesis through proteorhodopsin-generated proton motive force. Rhodobacteriaceae, Hyphomonadaceae, and Cytophagaceae genomes all contained carotenoid biosynthesis pathways, offering potential protection against free radicals generated during photosynthesis and/or exposure to ultraviolet radiation.

The GM7ARS4, GM202ARS2, and SP5GCR1 genomes all included a complete methylene-tetrahydromethanopterin dehydrogenase (dH4MPT)-dependent oxidation pathway, diagnostic for methylotrophic C-1 metabolism [39]. Each of these genomes also encoded a complete type III secretion system (T3SS), often associated with eukaryotic pathogenicity. No pathways for dH4MPT-dependent oxidation or T3SS biosynthesis were present in other genomes recovered from this study. Based on these data, the taxonomic relationship between GM7ARS4 and GM202ARS2, and the absence of any other previously described bacteria from the same order, we have provisionally assigned these two genomes to a new order named *Candidatus* Methylospongiales.

T3SS operons in both SP5GCR1 and the two Methylospongiales genomes contained 15–18 proteins annotated as T3SS components, including base, inner rod, and needle proteins; pore-forming translocation proteins; chaperonins; ATPases; and regulatory proteins. Although it was not possible to determine the nature of substrates being transported, T3SS operons also included matches to distinctive virulence-associated protein families (e.g., YscX), associated with human pathogens [40]. Placement of sponge microbiome YscR homologs in a reference tree of conserved, habitat-classified examples (Supplementary Figure S8, Additional file 1) [41] shows their closest relatives derive from extracellular bacteria associated with animal and insect hosts, but not plants, protists, or fungi. These results suggest possible roles for GM7ARS4, GM202ARS2, and SP5GCR1 in sponge host parasitism and/or pathogenicity. Genome sizes ranged from 1.6 to 1.8 Mbp, consistent with small sizes often observed in obligate symbionts and pathogenic bacteria [42–44].

Evidence identifying other community members as potential obligate symbionts was constrained by assembled genome incompleteness and limited availability of well-characterized free-living relatives. However, supporting evidence was provided in some cases by consistent presence or absence of clade-specific diagnostic features in multiple closely related MAGs from different samples. As an example, *Hormoscilla* genomes GUM202 and GUM007 have previously been shown to lack complete pathways for biotin synthesis [13]. This same deficiency was confirmed in *Hormoscilla* genomes from samples SP5, SP12, and GUM102, along with the presence of multiple transporters for importing this essential cofactor.

Genomic streamlining has previously been proposed as a shared feature of pelagic marine Rhodbacteriacae of the Roseobacter clade [45]. The genomes of GM7ARB1 and GM7ARB2, estimated to be 94–97% complete by CheckM analysis, are approximately 2.55 Mbp in size. This is smaller than the previously reported minimum genome size of 3.3 Mbp for Roseobacters, as well as closest free-living relative *Nioella nitratereducens* (4.0 Mbp). The four Rhodbacteriacae in the current study have 8–10% of their genomes devoted to transporter functions (Supplementary Table S6, Additional file 1), but lack genes for flagellar biosynthesis, and RuBisCO-mediated carbon fixation typically found in other family members. However, many other clade-specific characteristics have been preserved, including pathways for the synthesis of capsular polysaccharides and the degradation of phosphonates, urate, and dimethylsulfoniopropionate (DSMP).

Marine Bacteroidetes from the Cytophaga-Flavobacteria group are noted for their role in degrading organic matter during phytoplankton blooms, but group members containing proteorhodopsin have consistently smaller genomes than close taxonomic relatives lacking this gene function [46]. Consistent with these observations, Cytophagaceae genomes SP12BCY2, SP5BCY1, and GM202BCY1 (~ 3 Mbp) are similar in size to free-living family members containing proteorhodopsin, but smaller than closest sequenced relative *Ekhidna lutea* (4.2 Mbp), which does not*.* SP12BCY2, SP5BCY1, and GM202BCY1 are enriched in signal peptide-containing peptidases (9–12 per genome) and glycosidases (3–4 per genome), suggesting the retention of conserved heterotrophic capabilities for degrading extracellular proteins and polysaccharides (Supplementary Table S6, Additional file 1).

All three of these genomes have retained Bacteroidetes-specific gliding motility and Por (type IX) secretion system functions. The type IX secretion system is both an essential component of gliding motility in non-pathogenic species [47, 48] and a virulence factor in human and fish pathogens [49–51]. A large number of novel cadherin domain-containing proteins (Supplementary Table S6, Additional file 1), with closest GenBank matches at 29–44% amino acid identity, may facilitate adhesion to sponge hosts and/or particulate matter [52].

Potential symbiotic adaptations in Hyphomonadaceae genomes SP12HP1 and SP5AHP2 were suggested by the absence of both flagellar motility genes and the stalk formation pathway found in many other Hyphomonadaceae genomes [53]. However, genome sizes (2.4–2.5 Mbp) were only about 25% smaller than closest free-living relative *Hellea balneolensis* (3.2 Mbp). Lineage characteristic genes for heparinases, chondroitinases, and chitinases, and signal peptide-containing glycosidases were retained in SP12HP1 and SP5AHP2, potentially facilitating the degradation of host extracellular matrix as well as organic marine particulates. The presence of typical Hyphomonadaceae pathways for the transport and degradation of aromatic compounds such as benzoic acid suggests retention of diverse heterotrophic capabilities.

Rhodospirillales genomes SP5ARS3, GUM102ARS1, and GM202ARS1 have much smaller genomes than their closest sequenced relative, the terrestrial soil bacterium *Azospirillum brasilense* (< 3 versus 7.2 Mbp), but this difference could be due to expansion in *Azospirillum* rather than reduction in sponge-associated genomes. SP5ARS3, GUM102ARS1, and GM202ARS1 are unique among the genomes of this study in encoding pathways for carbon fixation via the Wood-Ljungdahl (Reductive Acetyl-CoA) pathway, the glyoxylate cycle, and trimethylamine degradation. The presence of nitrile hydratases and a large repertoire of transporters (9–12% of predicted coding sequences; Supplementary Table S6, Additional file 1) with predicted specificities for amino acids, oligopeptides, taurine, spermidine/putrescein, lipoproteins, ribose/xylose, and glycerol suggest metabolic versatility encompassing a wide variety of substrates, especially those containing amine groups. Genes encoding capsular biosynthesis may provide protection against host phagocytosis, viral attack, and/or hydrophobic toxins.

Marine relatives of genome GM7GCV1 include both free-living and host-associated species from Gammaproteobacteria family Cellovibronaceae, with AAI scores of 50% and 16S rRNA gene identities of 87–88%. All cultured examples have genome sizes of 4 Mbp or larger, including the dinoflagellate-associated *Halieaceae* LZ-16-2, GM7GCV1’s closest relative. The much smaller genome size of GM7GCV (1.8 Mbp) is typical for more distantly related, marine particle-associated Cellovibronaceae from the Tara Oceans project (e.g., TMED119 at AAI 45%; Supplementary Figure S3, Additional file 1), whose genome sizes range from 1.1 to 2.9 Mbp [54]. Factors responsible for smaller genome sizes in these uncultured, uncharacterized bacterial relatives are unknown.

The unavailability of sequenced genomes for relatives closer than order level makes it difficult to determine whether the SP5OBV1 genome lacks clade-specific features. However, it is unlikely that this species follows the same bacterivorous lifestyle as its distant cousins, not only because its genome size, estimated at 91% complete by CheckM analysis, is less than half as large as closest relative *Bdellovibrio bacteriovorus* (1.7 versus 3.8 Mbp), but also due to the absence of genes encoding flagellar motility and proteoglycan synthesis described as essential for this activity in other Bdellovibrionales species (reviewed in [55]). Fatty acid auxotrophy in SP5OBV1 is suggested by the absence of acetyl-CoA carboxylase and all other fatty acid biosynthesis pathway enzymes except FabG, coupled with the presence of complete pathways for lipoic acid metabolism and fatty acid degradation. Some mammalian pathogens are known to suppress endogenous biosynthesis while incorporating exogenous host fatty acids as a triclosan resistance mechanism, but previously reported genomic pathway deficiencies accompanying these adaptations have been much less extensive than those observed in SP5OVB1 [56].

### Viral sequences and phage defense

Electron microscopic studies have identified phage-like particles associated with *Lamellodysidea* sponge samples [57], but these have not yet been characterized by sequence analysis. In this study, viral scaffold candidates detected by VirSorter [58] in assembled holobiont metagenomes were dominated by GenBank matches to double stranded DNA tailed bacteriophage (Caudovirales) of the Siphoviridae, Myoviridae, and Podoviridae lineages (Supplementary Figure S9, Additional file 1). Matches to Microviridae, archaeal Bicaudaviridae, and eukaryotic Baculoviridae and Herpes viruses were also detected at low levels. Genetic heterogeneity of integrated phage regions can make them difficult to capture in metagenomic assemblies, potentially underestimating occurrence. However, abundant CRISPR sequences, transposons, and restriction enzymes roughly proportional to genome size in assembled MAGs suggest a past history of recurring viral challenges (Supplementary Table S7, Additional file 1).

Integrated prophage genomes sometimes carry passenger genes of bacterial origin that can modify the phenotype of the host, resulting in improved fitness of infected cells, a process known as lysogenic conversion [59]. *Lamellodysidea*-associated phage candidate scaffolds included bacterial genes encoding DNA methyltransferases, glucanases, peptidases, and vitamin B-12 biosynthesis. One candidate Myoviridae scaffold, from sample SP5, encoded two key enzymes (PhnI and PhnJ) of the carbon-phosphonate lyase pathway [60]. This pathway has previously been demonstrated to be enriched under phosphate limiting conditions in metagenomic marine microbes [61], and could potentially assist in liberating phosphate from recalcitrant organic particles.

### Secondary metabolite pathways

Secondary metabolite gene cluster candidates identified by antiSMASH [62] were most abundant in cyanobacterial taxa, with the largest number in previously described *Hormoscilla* genomes GM7CHS1 and GM202CHS1 (Supplementary Table S8, Additional file 1 [13];). *Hormoscilla* genomes SP12CHS1, SP5CHS1, and GM102CHS1 had fewer predicted clusters, but antiSMASH cluster detection sensitivity may have been reduced by scaffold fragmentation in these MAGs, which were assembled from Illumina reads without PacBio read supplementation.

The next most abundant source of biosynthetic gene clusters was *Prochloron* genome SP5CPC1, predicted to include two non-ribosomal peptide synthetases (NRPS), four terpene synthases, three ribosomally synthesized post-translationally modified peptides (RiPPs), and two flavin-dependent aromatic halogenases (Supplementary Table S8, Additional file 1). Although SP5CPC1 has a smaller genome size with fewer total clusters than previously sequenced *Prochloron* relatives [63], SP5CPC1 is also less complete, with short scaffolds and fragmented, incomplete pathway sequences that cannot easily be linked to specific molecular products. The aromatic halogenases were unrelated to those found in PBDE-producing strains of *Hormoscilla spongeliae* ([16], Supplementary Table S10B, Additional file 1), and putative RiPP clusters could only be classified as distantly related to non-cyanobactin bacteriocins and lassopeptides, based on HMM (Hidden Markov Model) pattern matches [62]. None of the putative RiPP clusters contained genes from the well-characterized patellamide pathway of *Prochloron didimei* [64].

Predicted biosynthetic clusters in non-cyanobacterial genomes were limited to terpenes and bacteriocin-related RiPPs, except for the SP12BCY1, SP5BCY1, and GUM202BCY1 genomes, which all contained an identical type III polyketide synthase (PKS). The closest database matches to this protein were from Cytophagaceae genera such as *Ekhidna*, *Marinoscillum*, and *Pontibacter* at 53–64% amino acid identity. Biosynthesis of plant-like flavonoids like those recently characterized in *Flavibacteria cheonhonense* [65] seems unlikely, because key pathway enzyme phenylalanine ammonia lyase is missing [66, 67]. However, type III PKS genes from the current study also matched CepAB genes from taxonomically distant sponge symbiont *Entotheonella gemina* (class Tectomicrobia) at 48% amino acid identity, suggesting phenolic lipids as a potential biosynthetic product [68].

Previously reported gene clusters encoding polybrominated compounds in assembled *Hormoscilla* genomes [13, 16] do not account for the full range of halogenated products described in *Lamellodysidea* sponges [69]. Additional diversity may be contributed by members of the broader microbial community, for example Hyphomonadaceae SP12AHP1 and SP5AHP2, which encode a highly expanded family of novel aromatic flavin-dependent halogenases (Supplementary Figure S10AB, Additional file 1). Several of these halogenases occur in sets of 2–4 tandem repeats, suggesting family expansion by gene duplication. Based on similarity to Pfam database model PF04820 [70], these proteins are annotated as flavin-dependent tryptophan halogenases. However, their closest match among experimentally characterized enzymes is the *brvH* gene product from *Brevundimonas* sp. BAL3, at 47% amino acid identity (Supplementary Figure S10B, Additional file 1). BrvH uses free indole rather than tryptophan as a substrate, preferentially incorporating bromine over chlorine into the C3 position [71].

It is possible that some of the additional diversity in halogenated compounds previously observed in *Lamellodysidea* sponges, including brominated phenols and catechols [72, 73], arises from degradative, rather than biosynthetic pathways. The SP12AHP1 and SP5AHP2 genomes each encode a complete 3,5-dichlorocatechol degradation pathway, characteristic of bacteria that use halogenated benzoates as sole carbon and energy sources [74]. Non-halogenated benzoate degradation pathways are common in other Hyphomonadaceae, but the SP12AHP1 and SP5AHP2 genomes are unique in encoding chlorocatechol 1,2-dioxygenase, a key enzyme that cleaves chlorocatechol rings to 2,4-dichloro-cis,cis-muconate, followed by spontaneous dehalogenation during further processing by muconate cycloisomerase, carboxymethylenebutenolidase, and maleylacetate reductase [75]. The closest chlorocatechol 1,2-dioxygenase match in the GenBank nr database (64% amino acid identity) was found in *Altererythrobacter marensis*, a free-living coastal marine bacterium from the Alphaproteobacteria order Sphingomonadales [76]. Dichlorocatechol degradation pathways in the SP12AHP1 and SP5AHP2 genomes are clustered together in the same conserved gene order as *A. marensis* (Supplementary Figure S11, Additional file 1).

Plasmids containing the 3,5-dichlorocatechol degradation pathway are frequently exchanged between environmental bacteria in chloroaromatic contaminated environments (reviewed in [77]). Although the pathway is not located on a plasmid or within a genomic island in SP12AHP1, SP5APH2, or *A. marensis*, patchy phylogenetic distribution and order-level relationships between closest protein sequence relatives suggest historical dissemination by horizontal gene transfer (Supplementary Figure 12, Additional file 1). It is not known whether amino acid sequence similarity to experimentally characterized chlorocatechol dioxygenases might also capture activity towards brominated substrates, including PBDEs, but this seems like a reasonable hypothesis for future testing.

## Discussion

Sponge-associated microbial communities have historically been classified into two broad categories, designated HMA (high microbial abundance, high diversity) and LMA (low microbial abundance, low diversity) [78]. Despite the fact that these legacy descriptions exclude situations where abundance and diversity might not be correlated, they are pervasive in the recent sponge microbiome literature, perhaps because physical quantification of microbial cell abundance in sponge tissues is technically challenging and rarely performed. High sponge seawater pumping rates have been proposed to promote aerobic, LMA communities, with lower rates favoring more anaerobic HMA communities [2]. These hypotheses are supported by results in the current study, where multiple samples from *Lamellodysidea herbacea* sponges, noted for high seawater pumping rates [3], contain a predominance of bacteria favoring aerobic rather than anaerobic metabolic pathways, with low overall community diversity.

The *Lamellodysidea herbacea* sponge microbiome has previously been characterized almost exclusively in terms of functional activities associated with its most dominant symbiont, *Hormoscilla spongeliae*. The results of this study reveal previously unreported potential metabolic activities, interactions, and community contributions of non-*Hormoscilla* species. Low microbial community complexity facilitated effective taxonomic binning and metagenomic assembly, producing long scaffolds enabling operon-context assessment of functional gene activities. The assembly of multiple population genomes from closely related taxa in different, independent samples helped establish core functional characteristics for novel sponge-associated groups previously known only from 16S rRNA gene fragments, and encountered only rarely in other sponge species.

The GM7ARS4 and GM202ARS2 genomes, classified in a new Alphaproteobacteria order (*Candidatus* Methylospongiales), and SP5GCR1, representing a new Gammaproteobacteria family, are, to the best of our knowledge, the first reported potential pathogens of *Lamellodysidea herbacea*, although type III secretion systems may also be associated with commensal or mutualistic relationships. The unlikely combination of methylotrophy with type III secretion systems consistently shared in distantly related taxa suggests potential exploitation of a common niche opportunity. Although no close relatives of these organisms have yet been reported in other sponges, the three near-complete genomes obtained in this study should enable more effective future searches based on predicted metabolic and physiological features.

The genomes sequenced in this study show strong evidence of adaptation to their sponge-host environment. Abundant light availability in the reef habitat fuels energy generation through three different processes, using phototrophic pigments with widely varying absorption maxima: chlorophyll *a* (660 nm), bacteriochlorphyll *a* (800 nm), and proteorhodopsin (~ 520 nm). UV damage protection is provided by carotenoid pigments. The possibility that photoactive pigments with differing optimal absorption spectra might provide advantages within micro-niches associated with different layers of sponge tissue would be an interesting topic for future studies.

High seawater flow rates induced by host pumping action create strong shear forces that could potentially dislodge microbiome inhabitants from sponge tissues. The expanded families of adhesive molecules and type IV pili found in many *Lamellodysidea*-associated bacteria would be expected to counteract these disruptive forces. Oxygen depleted by microbial respiration should be rapidly replenished from seawater flow and cyanobacterial photosynthesis, consistent with an environment that appears to favor organisms using aerobic processes for heterotrophic degradation of dissolved and particulate organics, and potentially discriminating against symbionts requiring more anaerobic conditions. Nitrogenase enzymes requiring the exclusion of oxygen were not observed. They may however be unnecessary in this environment due to a combination of host ammonia excretion and the release of fixed nitrogen from the degradation of organic compounds [2].

Previous bioenergetic analyses of microbial contributions to *Lamellodysidea* sponge nutrition have been limited to relatively simplistic models based on chlorophyll-mediated photosynthesis by a single microorganism. Functional pathway predictions and abundance data describing the new phototrophic and heterotrophic symbionts identified in this study should allow the expansion of these models to include contributions from multiple metabolic sources, encompassing a more accurate sampling of microbiome community diversity.

Microbial community compositions in all *Lamellodysidea* samples were strongly correlated with host clade taxonomy, whether assessed by metagenomic read recruitment or 16S rRNA gene analysis. The persistent co-occurrence of highly conserved Rhodobacteriaceae and Rhodospirillaceae species with *Hormoscilla* over an 11-year time period in Guam *Lamellodysidea* sponges (from 2005 in [14] to 2016 in the current work) suggests the likelihood of relatively stable, long-term host-microbe relationships. Previous analyses of Guam *Lamellodysidea* sponge microbiomes [14] failed to identify many of the taxa described in the current study, but examined 100-fold fewer 16S rRNA gene sequences, and did not include any representatives of host clades Ia or Ib. Further studies will be required to understand why host clade Ia contains some taxa, especially Hyphomonadaceae and *Prochloron*, that are absent in other clades, as well as why photoheterotrophic Rhodobacteriaceae were relatively abundant in clades Ia, Ib, and III, but nearly absent from clade II, where they were replaced by non-photosynthetic Rhodospirillaceae.

The biological functions and effects of locally concentrated PBDEs produced by *Hormoscilla* bacteria are unknown. Genomes SP12AHP and SP5AHP2 from clade Ia PBDE-producing sponges encode a potential mechanism for aerobic degradation of these compounds that may enable their use as nutritional energy sources. This proposed pathway includes a modified dioxygenase ring cleavage mechanism related to those used by other Hyphomonaceae for breaking down non-halogenated aromatic hydrocarbons.

A large number of biologically active secondary metabolite compounds have been isolated from *Lamellodysidea herbacea* sponges (reviewed in [79]), but distinguishing between those synthesized by the eukaryotic host versus microbial symbionts has proved challenging, especially in the absence of an assembled host genome. The unexpected discovery that the microbiomes of *Lamellodysidea herbacea* clade Ia sponges contain representatives of the prolific secondary metabolite-producer genus *Prochloron*, in addition to *Hormoscilla*, provides new opportunities to identify microbial sources for both previously characterized and undiscovered natural product molecules.

## Conclusions

This work has demonstrated sponge microbial community compositions to be both clade-specific and consistent over multi-year timescales. New insights have been provided into potential metabolic contributions of non-dominant members of *Lamellodysidea* microbial communities, encompassing energy generation through anoxygenic photosynthesis, methylotrophic C-1 metabolism, and aerobic heterotrophy of organic compounds, potentially including polybrominated diphenyl ethers produced by the dominant cyanobacterium, *Hormoscilla*. Genomic evidence suggests that both biosynthesis and degradation of the secondary metabolome may be linked among multiple taxa within the *Lamellodysidea* microbiome.

The reconstruction of high-quality MAGs representing all microbial taxa at relative abundances greater than 0.1% has enabled species-specific assignment of unique metabolic features that could not have been predicted from taxonomic data alone. This information will promote more representative models of marine invertebrate microbiome contributions to host bioenergetics and a better understanding of biosynthetic and degradative pathways for secondary metabolites and halogenated compounds in sponge-associated microbiota.

## Methods

### Sample collection and processing

Sponge samples used in this study, shown in Supplementary Table 1, Additional file 1, were collected between 2014 and 2016 from four different coastal sites in Guam. Frozen tissue samples were processed and prepared for DNA sequencing using Illumina HiSeq paired-end technology as described previously [13, 16]. Additional PacBio sequencing reads, obtained from cyanobacterial trichome enrichments of samples GUM7 and GUM202 [13], were used in the assembly of *Hormoscilla spongeliae* genomes from these samples. Sample host taxonomy was verified by amplification of ITS primer regions from bulk DNA, according to the protocol described in [80].

16S rRNA gene amplification was performed using V4-5 region primers 515F-4Y (5′-GTG**Y**CAGCMGCCGCGGTAA) and 926R (5′-CCGYCAATTYMTTTRAGTTT), as previously described [81]. Paired-end reads were generating using Illumina MiSeq v3 (2 × 300 bp) sequencing. DADA2 v 1.12 [82] was used within QIIME2 [83] to trim, denoise, merge, remove chimeras, and cluster the assembled reads, with the following program parameters: --p-trunc-len-f 250 --p-trunc-len-r 180 --p-trim-left-f 20 --p-trim-left-r 20 --p-max-ee-f 3 --p-max-ee-r 5. Taxonomic classifications were assigned based on the SILVA v132 reference database [84], supplemented with 19 near-complete 16S rRNA genes obtained from targeted metagenomic assemblies in this study.

### Metagenomic assembly

Illumina HiSeq 2500 reads (2 × 100 or 2 × 150 PE) from metagenomic DNA libraries were quality filtered and trimmed using Trimmomatic version 0.339 [85]. Preliminary guide assemblies were created using IDBA-UD version 1.1.117 [86] with default parameters, followed by mapping of input reads to scaffolds using the end-to-end option of Bowtie2, version 2.218 [87]. Coverage depth and percentage of singleton versus paired reads were calculated from output of the idxstats module from samtools version 0.1.191 [88]. Preliminary scaffold bins were assigned based on percent GC, nucleotide composition, coverage depth, and taxonomic classification by DarkHorse version 2.0 [89, 90], as previously described [91]. Read subsets were supplemented as necessary to include both R1 and R2 members of each constituent read pair, then re-assembled.

Second round assemblies were performed using both IDBA-UD, and, alternatively Celera Assembler version 8.3 [92], configured with merSize = 17, utgGenomeSize = 5 Mb, and utgErrorRate = 0.01. Second round assembly scaffolds were quality-screened by re-applying the same methods used for initial taxonomic binning (percent GC, coverage depth, and DarkHorse taxonomic classification), with an added requirement that each scaffold should contain more than 90% paired (versus singleton) reads. Final assembly completeness and contamination were assessed using CheckM version 1.07 with the default set of bacterial marker genes [24].

*Hormoscilla* genome assemblies have been previously reported for samples GUM202 and GUM007 using a combination of Illumina and PacBio reads from trichome-enriched preparations [13]. In the current study, PacBio reads from sample GUM007 were re-assembled without Illumina read supplementation using CANU version 1.8 with default parameters [93], which greatly improved the GUM007 *Hormoscilla* assembly (100% versus 94.5% completeness, 1.8% versus 2.2% contamination, and 6 versus 64 contigs). This approach could not, however, be applied to the GUM202 *Hormoscilla* genome, because the smaller number of available PacBio reads (31,034 versus 180,838) failed to provide sufficient coverage.

### Phylogenetic placement

16S rRNA gene sequences for closely related species were obtained by blastn searches against SILVA database release 132 [94], and aligned with sequences extracted from assembled genomes using the SILVA Incremental Aligner (SINA) version 1.2.11 [95]. 16S rRNA gene trees were constructed from these alignments using FastTree version 2.1.8 [96] and visualized with FigTree version 1.4.3 [97]. Multi-locus gene trees were constructed using PhyloPhlAn version 0.99 [98]. Average amino acid identities were calculated using the online AAI-Matrix Genome-based distance matrix calculator [26].

### Environmental database searches

16S rRNA genes identified in metagenome-assembled genomes (MAGs) from this study were compared to reference database sets by blastn searches, with a minimum cutoff alignment length of 360 nucleotides and maximum *e* value of 1e−7. Quantitative abundances of blastn matches to OTUs from samples in the Sponge Microbiome Database were obtained by querying a custom MySQL database, constructed by reformatting Supplementary files S3, S4, and S5 from [30] into the schema shown in Supplementary Figure 13, Additional file 1.

### Functional annotation

All MAGs generated in this study were annotated at IMG-MER [99]. CRISPR repeat regions were identified using the MinCED (Mining CRISPRs in Environmental Datasets) program, version 0.3.0 [100]. Potentially over-represented protein functional families were identified using Hidden Markov Models (HMMs) from the Pfam-A version 32 [70] and TIGRFAM release 15 [101] databases. In cases where models with overlapping functional activities matched the same target protein, only the HMM with the highest bitscore was included in quantitative tallies, so that no protein was counted more than once. Candidate viral sequences were identified using VirSorter version 1.0.5 [58] and taxonomically classified using DarkHorse version 2.0 with a custom reference database consisting of all viral sequences in the GenBank nr database as of January 2, 2019. Biosynthetic clusters encoding potential secondary metabolites were identified using AntiSMASH version 5.0 [62].

## Supplementary information

**Additional file 1: Supplementary Figure 1**. Cyanobacteria MAGs classified taxonomically. A) PhyloPhlAn multi-locus concatenated tree, with *Crinalium epipsammum* as an outgroup. B) 16S rRNA gene/and average amino acid identity matrix with closest database relatives. Guidelines for assigning species, genus, family, and order-level taxonomic granularity were based on [25, 26] for AAI and [38] for 16S rRNA gene percent nucleotide identity. **Supplementary Figure 2**. Bacteroidetes MAGs classified taxonomically. A) PhyloPhlAn multi-locus concatenated tree, with SP5OBV1 as an outgroup. B) 16S rRNA gene/and average amino acid identity matrix with closest database relatives. **Supplementary Figure 3.** Alphaproteobacteria MAGs classified taxonomically. A) PhyloPhlAn multi-locus concatenated tree, with SP5OBV1 as an outgroup. B) 16S rRNA gene/and average amino acid identity matrix with closest database relatives. **Supplementary Figure 4**. Gammaproteobacteria MAGs classified taxonomically. A) PhyloPhlAn multi-locus concatenated tree, with SP5OBV1 as an outgroup. B) 16S rRNA gene/and average amino acid identity matrix with closest database relatives. **Supplementary Figure 5**. Oligoflexia MAG classified taxonomically. A) PhyloPhlAn multi-locus concatenated tree, with *Oceanobaculum indicum* as an outgroup. B) 16S rRNA gene/and average amino acid identity matrix with closest database relatives. **Supplementary Figure 6.** Relative abundance of metagenomically assembled sequences in 16S rRNA gene amplicon data sets. Amplified 16S rRNA gene sequences were recruited by blastn search at 97% identity to metagenomically assembled 16S rRNA genes and pooled into taxonomic groups as shown in Figure 3. Relative abundances in this chart are normalized to compensate for differences in total number of 16S rRNA reads obtained for each sponge sample (see Supplementary table 2 for total numbers of cleaned, merged amplicon reads). MAGs GM102CHS1, GM7ARS4, SP5ARS3, GM102ARS1 could not be included in this table because they did not contain any sequences overlapping the amplified region of the 16S rRNA gene. **Supplementary Figure 7.** Relative abundance of raw metagenomic reads mapping to assembled MAGS. Percentages are based on Bowtie read recruitment at 100% identity. MAGs were pooled into taxonomic groups as shown in Figure 3. A) Relative abundances for all classified reads; B) read recruitment percentages, including unclassified reads. All percentages have normalized for number of reads per sample, but not adjusted for potential differences in genome size. **Supplementary Figure 8**. Host associations of Type III secretion protein YscR homologs. A) Highlighted YscR homologs from MAGs of this study are shown in the context of a phylogenetic tree annotated with host types as described in [41]. B) Position of YscR homolog in *Yersinia* Type III secretion structure, adapted from [103]. **Supplementary Figure 9**. VirSorter candidate scaffolds in holobiont assemblies. Virus taxonomic groups were obtained by DarkHorse assignment based on blastx matches to a custom database containing all viral sequences in the GenBank nr database as of January 2, 2019. **Supplementary Figure 10**. Flavin-dependent aromatic halogenases from SP12AHP1 and SP5AHP2. A) Operon structure map from IMG-MER [102] showing relative positions of tandem repeats in red. B) Phylogenetic tree of protein sequences, showing positions of putative halogenase sequences from this study relative to experimentally characterized database examples described in [71]. The tree is rooted with the bmp5 gene of *Pseudoalteromonas putida* [104]. PBDE pathway halogenases from *Hormoscilla* strains SP5, SP12, GUM102, and GUM202 clade with the bmp5 outgroup. **Supplementary Figure 11**. Comparison of candidate haloaromatic degradation gene neighborhoods. A) Gene neighborhood maps with halocatechol dioxygenase genes indicated in red. Maps and tables of predicted genes for B) SP12AHP1, C) SP5AHP2 and D) *Altererythrobacter marensis* were obtained using the automated IMG-ER annotation pipeline [102]. **Supplementary Figure 12**. Phylogenetic tree of halocatechol 1,2 dioxygenases. Tree shows SP12AHP1 and SP5AHP1 halocatechol 1,2-dioxygenases, highlighted in red, compared to closest GenBank nr database protein relatives. Chemically characterized chlorocatechol 1,2-dioxygenases are highlighted in blue. **Supplementary Figure 13**. MySQL schema for extracting sample metadata and abundance statistics from the Sponge Microbiome Database. Tables were populated using data in Supplementary tables 3,4, and 5 from [30]. **Supplementary Table 1**. *Lamellodysidea* sponge sampling metadata. **Supplementary Table 2**. Sponge holobiont and trichome metagenomic assembly statistics. Note: *GUM202 trichome enrichment reads were assembled in combination with Illumina GUM202 holobiont sequences, as previously described in [13]. **Supplementary Table 3**. MIMAG parameters for assembled genomes. **Supplementary Table 4.** Closest environmental matches from the GenBank nr database to 16S rRNA genes obtained from MAGs. GM102CHS1, GM7ARS4, SP5ARS3, GM102ARS1 are not included in this table because they did not include any 16S rRNA genes of 300 nt or longer. **Supplementary Table 5**. Assembled MAG 16S rRNA gene matches to Sponge Microbiome Database samples at 97% nucleotide identity. Database sequences were 99 bp segments amplified from the V4 region of the 16S rRNA gene [30]. Sponge Microbiome Database samples with the maximum number of matches to each MAG-associated 16S rRNA gene from this study were selected to determine the number and percent of matched sequences, and reported here along with host genus and geographical collection site. GM102CHS1, GM7ARS4, SP5ARS3, GM102ARS1 are not included in this table because they did not include any sequences overlapping the V4 region of the 16S rRNA gene. **Supplementary Table 6.** Tally of membrane-associated gene products in MAGs. Values for adhesive genes, signal sequences, and transporter types were tallied based on keyword searches of MAG annotations at IMG-MER [102]. **Supplementary Table 7.** Viral defense genes in assembled MAGs. Values were tallied based on keyword searches of MAG annotations at IMG-MER [102]. **Supplementary Table 8.** Biosynthetic gene clusters predicted by AntiSmash [62], supplemented with aromatic halogenases predicted by Pfam database model PF04820 [70].

## Data Availability

Sequence datasets generated and/or analyzed during the current study (sequencing reads, genome assemblies, and amplified ITS and 16S rRNA sequences) are available in the NCBI GenBank repository under BioProject ID PRJNA320446, and the IMG-MER database [102] under genome accession numbers 2643221529, 2747842407, 2758568580, 2758568581, 2767802581, 2808606420-34, 2814122959, 2830827416, and 3300008641.

## Abbreviations

PBDE: Polybrominated diphenyl ether
ITS: Internal transcribed spacer
Mbp: Megabase pair
nt: Nucleotide
MAG: Metagenome-assembled genome
TCA: Tricarboxylic acid
NRPS: Non-ribosomal peptide synthase
HMM: Hidden Markov Model
PKS: Polyketide synthase
