# Supplementary material for: A genomic view of trophic and metabolic diversity in clade-specific Lamellodysidea sponge microbiomes
Source: Microbiome. 2020 Jun 23;8:97. doi: 10.1186/s40168-020-00877-y (PMC7313196; doi:10.1186/s40168-020-00877-y)
Supplement: Supplementary file 2 — Additional file 1: Supplementary Figure 1. Cyanobacteria MAGs classified taxonomically. A) PhyloPhlAn multi-locus concatenated tree, with Crinalium epipsammum as an outgroup. B) 16S rRNA gene/and average amino acid identity matrix with closest database relatives. Guidelines for assigning species, genus, family, and order-level taxonomic granularity were based on [25, 26] for AAI and [38] for 16S rRNA gene percent nucleotide identity. Supplementary Figure 2. Bacteroidetes MAGs classified taxonomically. A) PhyloPhlAn multi-locus concatenated tree, with SP5OBV1 as an outgroup. B) 16S rRNA gene/and average amino acid identity matrix with closest database relatives. Supplementary Figure 3. Alphaproteobacteria MAGs classified taxonomically. A) PhyloPhlAn multi-locus concatenated tree, with SP5OBV1 as an outgroup. B) 16S rRNA gene/and average amino acid identity matrix with closest database relatives. Supplementary Figure 4. Gammaproteobacteria MAGs classified taxonomically. A) PhyloPhlAn multi-locus concatenated tree, with SP5OBV1 as an outgroup. B) 16S rRNA gene/and average amino acid identity matrix with closest database relatives. Supplementary Figure 5. Oligoflexia MAG classified taxonomically. A) PhyloPhlAn multi-locus concatenated tree, with Oceanobaculum indicum as an outgroup. B) 16S rRNA gene/and average amino acid identity matrix with closest database relatives. Supplementary Figure 6. Relative abundance of metagenomically assembled sequences in 16S rRNA gene amplicon data sets. Amplified 16S rRNA gene sequences were recruited by blastn search at 97% identity to metagenomically assembled 16S rRNA genes and pooled into taxonomic groups as shown in Figure 3. Relative abundances in this chart are normalized to compensate for differences in total number of 16S rRNA reads obtained for each sponge sample (see Supplementary table 2 for total numbers of cleaned, merged amplicon reads). MAGs GM102CHS1, GM7ARS4, SP5ARS3, GM102ARS1 could not be included in this table becau [file 40168_2020_877_MOESM1_ESM.pdf]

Supplementary Information

A genomic view of trophic and metabolic diversity in clade-specific *Lamellodysidea* sponge microbiomes

Sheila Podell, Jessica M. Blanton, Aaron Oliver, Michelle A. Schorn, Vinayak Agarwal, Jason S. Biggs, Bradley S. Moore, Eric E. Allen

Contents

Supplementary Figures S1-S13 2

Supplementary Tables S1-S8 16

References 26

Supplementary Figure 1. Cyanobacteria MAGs classified taxonomically. A) PhyloPhlAn multi-locus concatenated tree [1], with *Crinalium epipsammum* as an outgroup. B) 16S rRNA gene/and average amino acid identity matrix with closest database relatives. Guidelines for assigning species, genus, family, and order-level taxonomic granularity were based on [2, 3] for AAI and [4] for 16S rRNA gene percent nucleotide identity.

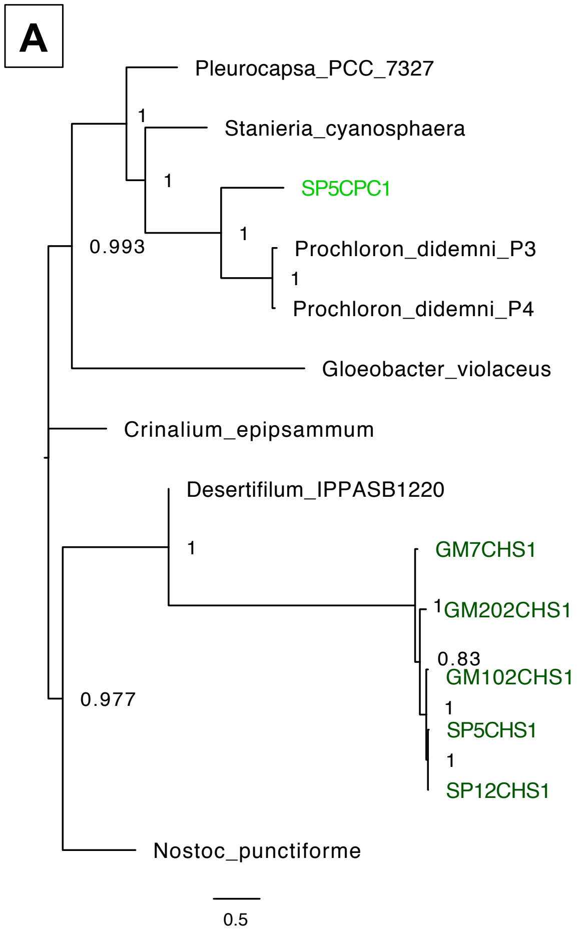

B

Stanieria_cyanosphaera	SP5CPC	Prochloron_didemni_P3	Prochloron_didemni_P4	Gloeobacter_violaceus	Crinalium_epipsammum	Desertifilum_IPPASB1220	GM7CHS1	GM202CHS1	GM102CHS1	SP12CHS1	SP5CHS1	Nostoc_punctiforme	
92/65	89/61	89/61	89/61	88/51	90/63	91/62	90/60	90/60	-/60	89/60	90/60	88/63	Pleurocapsa_PCC_7327
	92/59	93/60	92/61	88/49	90/60	91/60	91/58	90/58	-/59	90/58	91/58	89/60	Stanieria_cyanosphaera
		95/69	94/69	88/47	89/57	90/58	92/58	91/58	-/57	92/58	92/57	90/57	SP5CPC
			100/97	89/48	90/58	90/58	92/58	91/58	-/58	92/58	92/58	89/59	Prochloron_didemni_P3
				88/48	89/59	90/58	91/59	90/59	-/59	91/59	91/59	89/59	Prochloron_didemni_P4
					88/50	88/51	89/49	88/49	-/49	88/50	88/49	87/52	Gloeobacter_violaceus
						91/63	90/61	89/61	-/61	89/61	89/61	90/66	Crinalium_epipsammum
							92/64	90/64	-/64	90/64	90/64	90/63	Desertifilum_IPPASB1220
								98/93	-/92	99/92	99/92	90/61	GM7CHS1
									-/92	98/93	98/92	90/61	GM202CHS1
										-/97	-/97	-/62	GM102CHS1
											100/99	90/62	SP12CHS1
												90/61	SP5CHS1

Species
16S >98.8, AAI 95-100

Genus
16S 95-98.6, AAI 65-95

Family
16S 92-95, AAI 45-65

Ambiguous
Family/Order

Supplementary Figure 2. Bacteroidetes MAGs classified taxonomically. A) PhyloPhlAn multi-locus concatenated tree [1], with SP5OBV1 as an outgroup. B) 16S rRNA gene/and average amino acid identity matrix with closest database relatives.

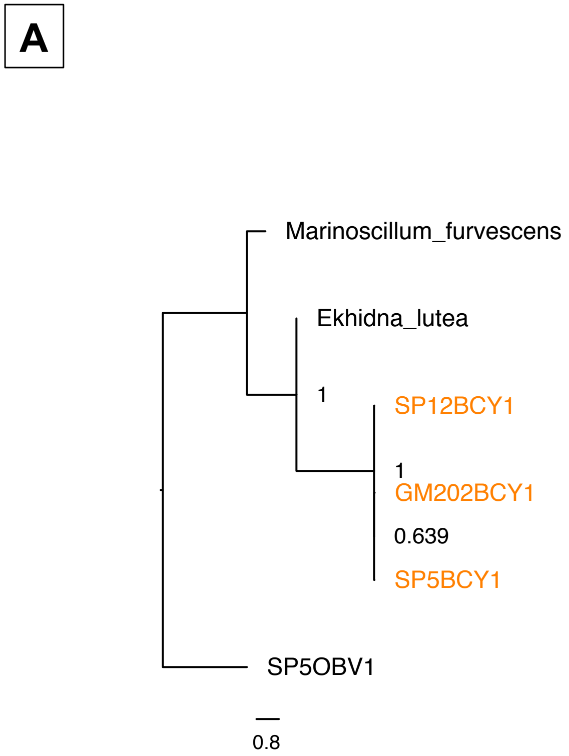

B

	GM202BCY1	SP12BCY1	SP5BCY1	Marinoscillum_furvescens
Ekhidna_lutea	95/67	95/67	95/67	92/57
GM202BCY1		100/98	100/98	91/57
SP12BCY1			100/99	91/57
SP5BCY1				91/57

Species
16S >98.8, AAI 95-100

Genus
16S 95-98.6, AAI 65-95

Family
16S 92-95, AAI 45-65

Ambiguous
Family/Order

Supplementary Figure 3. Alphaproteobacteria MAGs classified taxonomically. A) PhyloPhlAn multi-locus concatenated tree [1], with SP5OBV1 as an outgroup. B) 16S rRNA gene/and average amino acid identity matrix with closest database relatives.

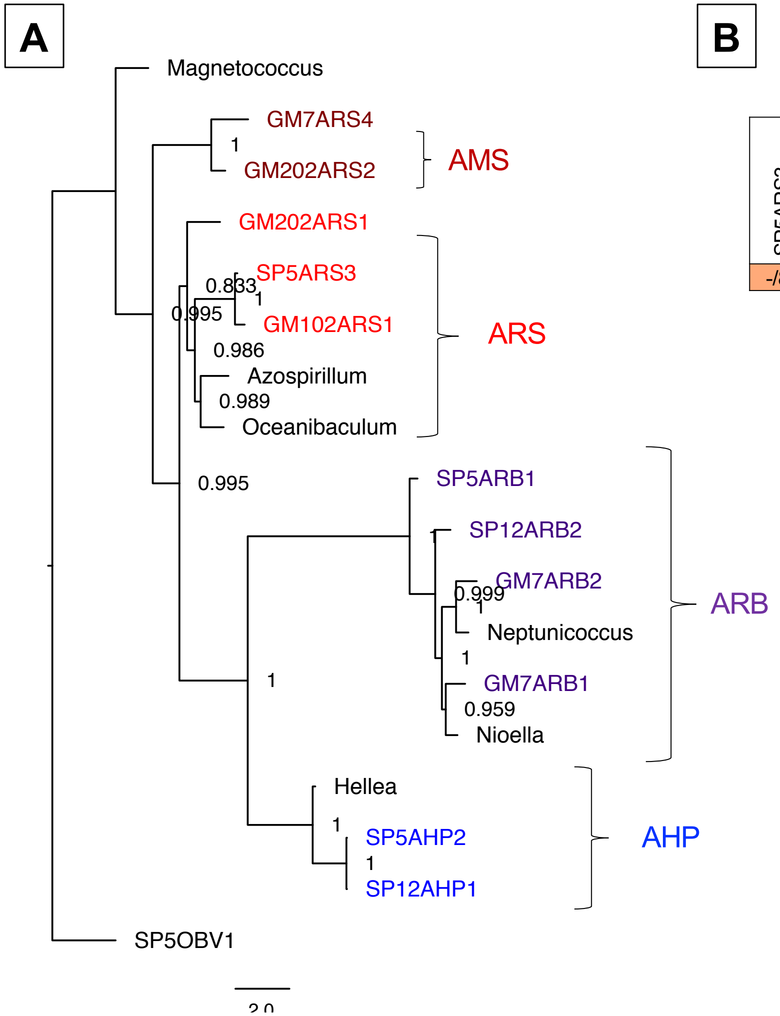

B

SP5ARS3	GM102ARS1	GUM202ARS1	GM7ARS4	GM202ARS2	SP5AHP2	SP12AHP1	Hellea	SP5ARB1	SP12ARB2	Nioella	GM7ARB1	Neptunicoccus	GM7ARB2	
-/87	-/49	86/50	-/41	84/41	86/41	86/42	88/42	89/45	88/45	86/47	86/46	86/46	89/45	Azospirillum
	-/51	-/52	-/44	-/44	-/45	-/45	-/45	-/47	-/48	-/48	-/48	-/48	-/48	SP5ARS3
		-/51	-/44	-/44	-/45	-/45	-/45	-/46	-/47	-/48	-/48	-/48	-/48	GM102ARS1
			-/44	88/44	86/45	86/45	87/44	84/46	89/45	88/47	87/48	87/47	86/46	GUM202ARS1
				-/55	-/41	-/41	-/41	-/41	-/41	-/42	-/42	-/42	-/41	GM7ARS4
					86/41	86/41	87/41	86/41	88/41	88/41	86/42	84/41	88/41	GM202ARS2
						100/99	95/61	85/44	84/44	88/45	89/45	88/45	87/45	SP5AHP2
							95/61	85/44	84/44	88/45	89/46	88/45	87/45	SP12AHP1
								85/44	84/43	88/45	88/45	88/45	88/45	Hellea
									98/59	87/59	85/59	85/56	83/56	SP5ARB1
										88/59	83/60	88/56	82/57	SP12ARB2
											95/66	93/60	89/58	Nioella
												92/58	87/60	GM7ARB1
													88/65	Neptunicoccus

Species
16S >98.8, AAI 95-100

Genus
16S 95-98.6, AAI 65-95

Family
16S 92-95, AAI 45-65

Ambiguous
Family/Order

Supplementary Figure 4. Gammaproteobacteria MAGs classified taxonomically. A) PhyloPhlAn multi-locus concatenated tree [1], with SP5OBV1 as an outgroup. B) 16S rRNA gene/and average amino acid identity matrix with closest database relatives.

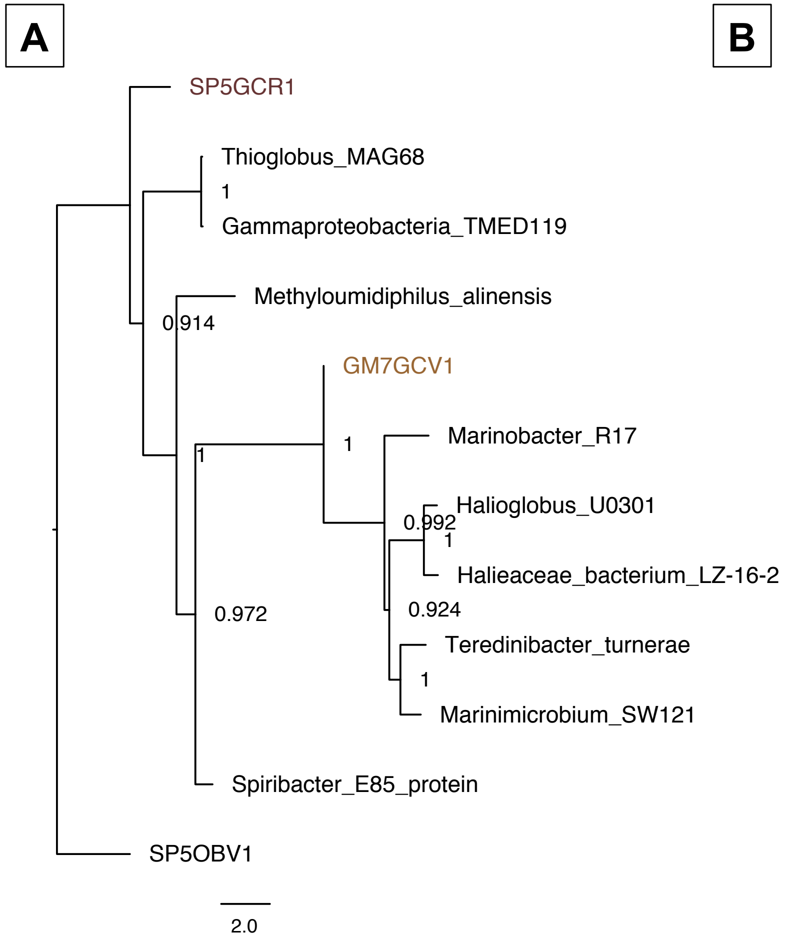

B

Halieaceae_LZ-16-2	Halioglobus_HI00S01	Marinimicrobium_SW121	Marinobacter_R17	SP5GCR1	TMED119	Thioglobus_MAG68	Spiribacter_E85	
-/50	87/50	88/50	88/48	85/43	-/45	-/45	86/47	GM7GCV1
	-/65	-/52	-/50	-/44	-/46	-/46	-/48	Halieaceae_LZ-16-2
		91/51	88/50	86/43	-/46	-/47	-/47	Halioglobus_HI00S01
			91/51	87/43	-/46	-/46	48	Marinimicrobium_SW121
				88/44	-/46	-/47	49	Marinobacter_R17
					-/48	-/49	87/45	SP5GCR1
						-/97	-/48	TMED119
							-/48	Thioglobus_MAG68

Species
16S >98.8, AAI 95-100

Genus
16S 95-98.6, AAI 65-95

Family
16S 92-95, AAI 45-65

Ambiguous
Family/Order

Supplementary Figure 5. Oligoflexia MAG classified taxonomically. A) PhyloPhlAn multi-locus concatenated tree [1], with *Oceanobaculum indicum* as an outgroup. B) 16S rRNA gene/and average amino acid identity matrix with closest database relatives.

A

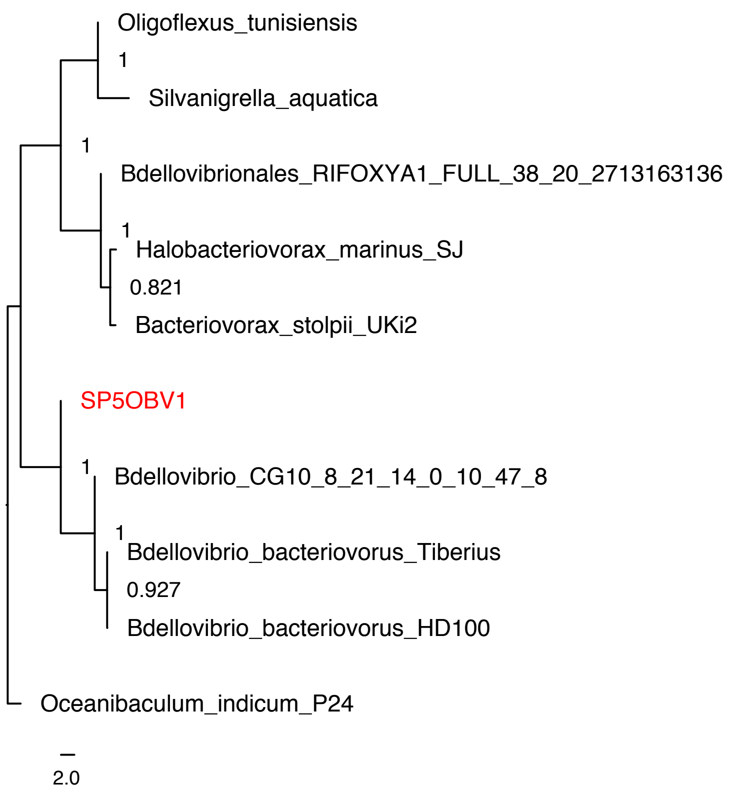

B

Silvanigrella_aquatica	Bdellovibrionales_RIFOXYA1	Halobacteriovorax_marinus_SJ	Bacteriovorax_stolpii_UKi2	SP5OBV1	Bdellovibrio_CG10	Bdellovibrio_bacteriovorus_Tiberius	Bdellovibrio_bacteriovorus_HD100	
84/37	-/35	86/36	88/37	85/36	86/38	88/38	88/38	Oligoflexus_tunisiensis
	-/35	88/36	86/36	85/36	86/37	86/37	86/37	Silvanigrella_aquatica
		-/46	-/45	-/36	-/36	-/36	-/36	Bdellovibrionales_RIFOXYA1
			89/50	85/37	85/38	85/38	86/38	Halobacteriovorax_marinus_SJ
				86/36	87/39	88/40	88/40	Bacteriovorax_stolpii_UKi2
					88/40	85/40	83/40	SP5OBV1
						90/54	90/54	Bdellovibrio_CG10
							99/94	Bdellovibrio_bacteriovorus_Tiberius

Species
16S >98.8, AAI 95-100

Genus
16S 95-98.6, AAI 65-95

Family
16S 92-95, AAI 45-65

Ambiguous
Family/Order

Supplementary Figure 6. Relative abundance of metagenomically assembled sequences in 16S rRNA gene amplicon data sets. Amplified 16S rRNA gene sequences were recruited by blastn search at 97% identity to metagenomically assembled 16S rRNA genes and pooled into taxonomic groups as shown in Figure 3. Relative abundances in this chart are normalized to compensate for differences in total number of 16S rRNA reads obtained for each sponge sample (see Supplementary table 2 for total numbers of cleaned, merged amplicon reads). Metagenomically assembled genomes GM102CHS1, GM7ARS4, SP5ARS3, and GM102ARS1 could not be included in this table because they did not contain any sequences overlapping the amplified region of the 16S rRNA gene.

group	SP12	SP5	SP1	SP8	GUM038	GUM040	GUM058	GUM096	GUM204		GUM020	GUM102	GUM203		GUM201	GUM202		GUM069	GUM007		seawater
CHS	28%	46%	36%	20%	38%	26%	24%	36%	67%		47%	41%	24%		51%	57%		47%	46%		0.1%
CPC	40%	5%	7%	1%	7%	0%	21%	10%	0%		0%	0%	32%		0%	0%		0%	0%		0.0%
ARB	2%	4%	7%	2%	7%	2%	7%	4%	1%		2%	8%	9%		1%	0%		10%	11%		0.0%
GCR	3%	1%	5%	2%	3%	5%	3%	4%	8%		22%	14%	5%		0%	0%		0%	0%		0.0%
AHP	4%	9%	1%	6%	9%	13%	5%	8%	0%		0%	0%	5%		0%	0%		0%	0%		0.0%
ARS	0%	0%	0%	0%	0%	0%	0%	0%	0%		0%	0%	0%		28%	21%		0%	0%		0.0%
BCY	4%	1%	6%	7%	2%	2%	2%	3%	0%		0%	0%	1%		7%	4%		0%	0%		0.0%
GCV	0%	0%	0%	0%	0%	0%	0%	0%	0%		0%	0%	0%		0%	0%		12%	12%		0.0%
AMS	1%	1%	2%	0%	1%	1%	2%	1%	0%		0%	0%	1%		1%	1%		0%	0%		0.0%
OBV	0%	0%	0%	0%	0%	0%	0%	0%	0%		1%	0%	0%		0%	0%		0%	0%		0.0%
Total	82%	68%	62%	39%	67%	48%	62%	65%	76%		72%	63%	76%		88%	82%		70%	69%		0.1%

Supplementary Figure 7. Relative abundance of raw metagenomic reads mapping to assembled MAGS. Percentages are based on Bowtie read recruitment at 100% identity. MAGs were pooled into taxonomic groups as shown in Figure 3. A) Relative abundances for all classified reads; B) read recruitment percentages, including unclassified reads. All percentages have normalized for number of reads per sample, but not adjusted for potential differences in genome size.

A

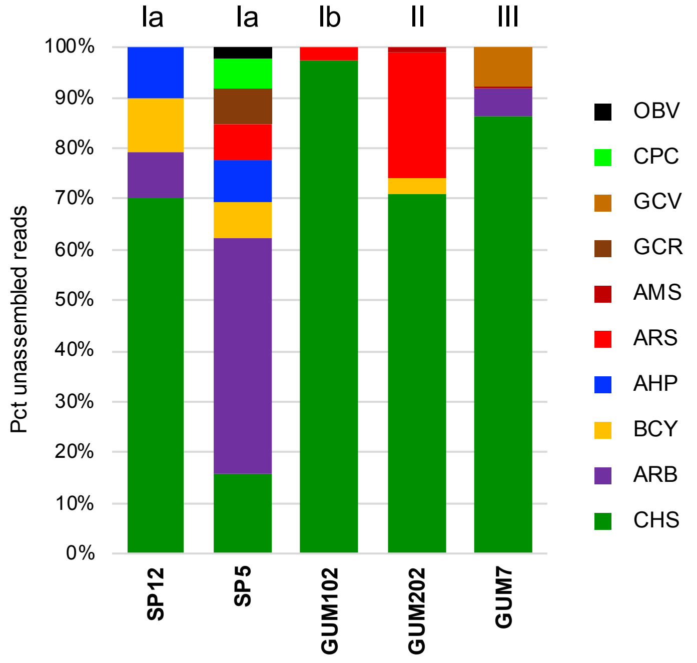

B

	SP12	SP5	GUM102	GUM202	GUM7
CHS	7.1%	3.6%	6.2%	12.9%	37.6%
ARB	0.9%	10.8%			2.4%
BCY	1.1%	1.6%		0.6%	
AHP	1.0%	1.9%			
ARS		1.6%	0.2%	4.5%	
GCV					3.4%
GCR		1.6%			
AMS				0.2%	0.1%
OBV		0.5%			
CPC		1.4%			
other	89.9%	76.9%	93.7%	81.8%	56.4%

Supplementary Figure 8. Host associations of Type III secretion protein YscR homologs. A) Highlighted YscR homologs from MAGs of this study are shown in the context of a phylogenetic tree annotated with host types as described in [5]. B) Position of YscR homolog in *Yersinia* Type III secretion structure, adapted from [6].

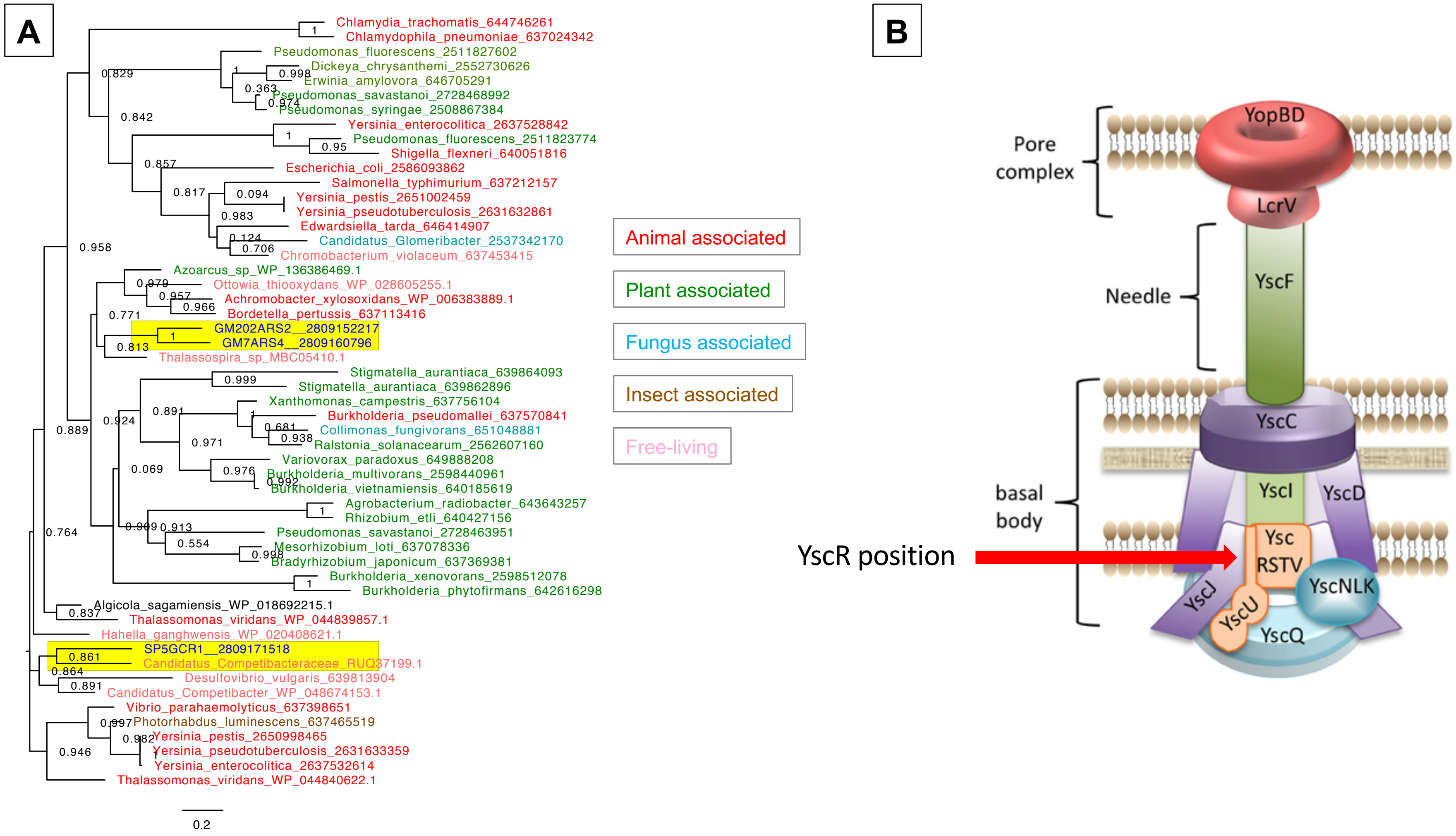

Supplementary Figure 9. VirSorter candidate scaffolds in holobiont assemblies. Virus taxonomic groups were obtained by DarkHorse assignment based on blastx matches to a custom database containing all viral sequences in the GenBank nr database as of Jaunuary 2, 2019.

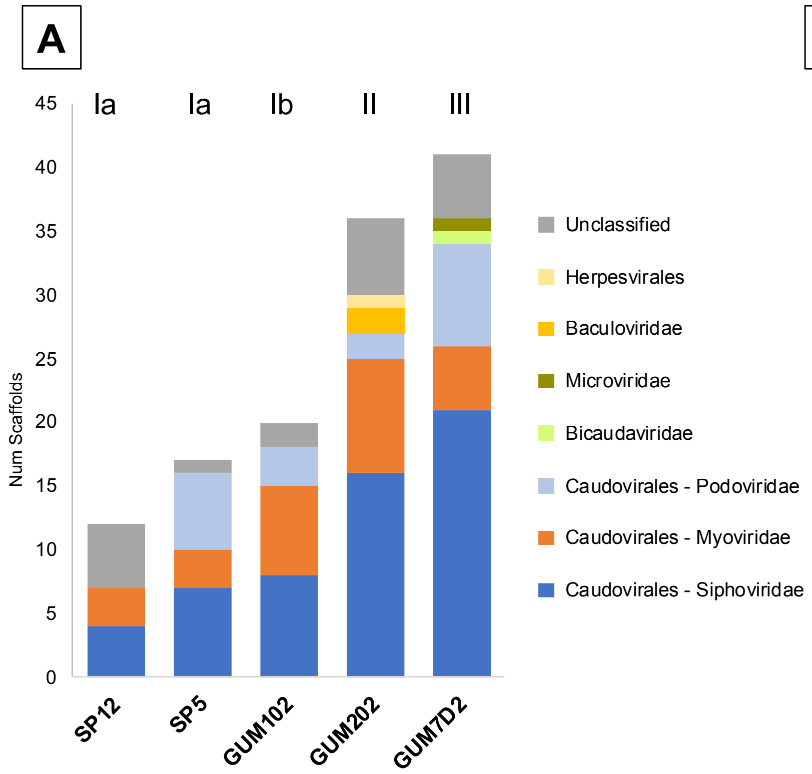

B

**sample**	**Total assembled scf > 3K**	**Virsorter candidate scf > 3K**	**Non-prophage candidates**	**Prophage candidates**
SP12	9,939	7	7	0
SP5	5,437	17	14	3
GUM102	16,484	20	17	3
GUM202	34,352	36	31	5
GUM7D2	30,973	42	36	6

Supplementary Figure 10. Flavin-dependent aromatic halogenases from SP12AHP1 and SP5AHP2. A) Operon structure map from IMG-MER [7] showing relative positions of tandem repeats in red. B) Phylogenetic tree of protein sequences, showing positions of putative halogenase sequences from this study relative to experimentally characterized database examples described in [8]. The tree is rooted with the bmp5 gene of *Pseudoalteromonas putida* [9]. PDBE pathway halogenases from *Hormoscilla* strains SP5, SP12, GUM102, and GUM202 clade with the bmp5 outgroup.

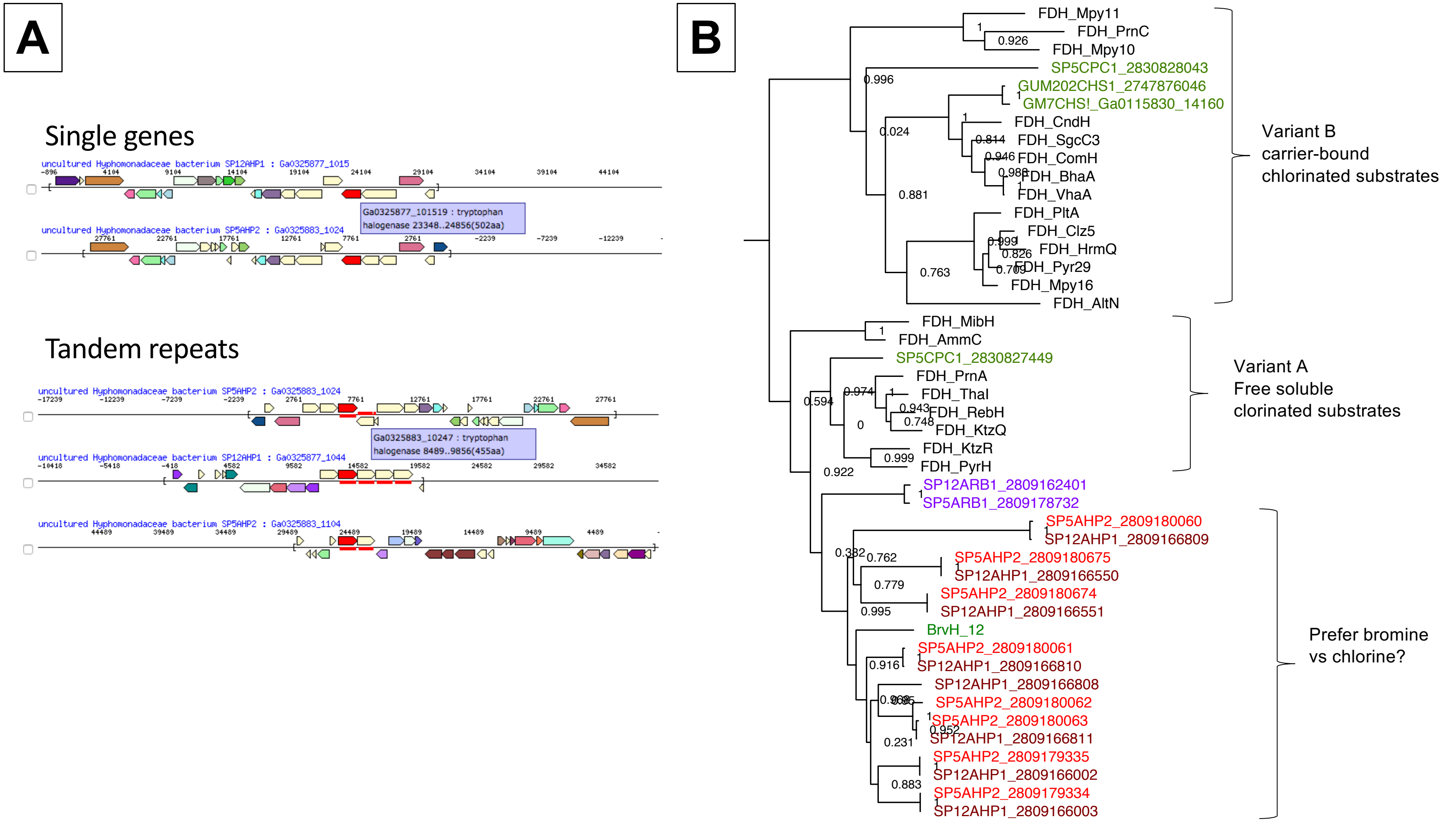

Supplementary Figure 11. Comparison of candidate haloaromatic degradation gene neighborhoods. A) Gene neighborhood maps with halocatechol dioxygenase genes indicated in red. Maps and tables of predicted genes for B) SP12AHP1, C) SP5AHP2 and D) *Altererythrobacter marensis* were obtained using the automated IMG-ER annotation pipeline [7]

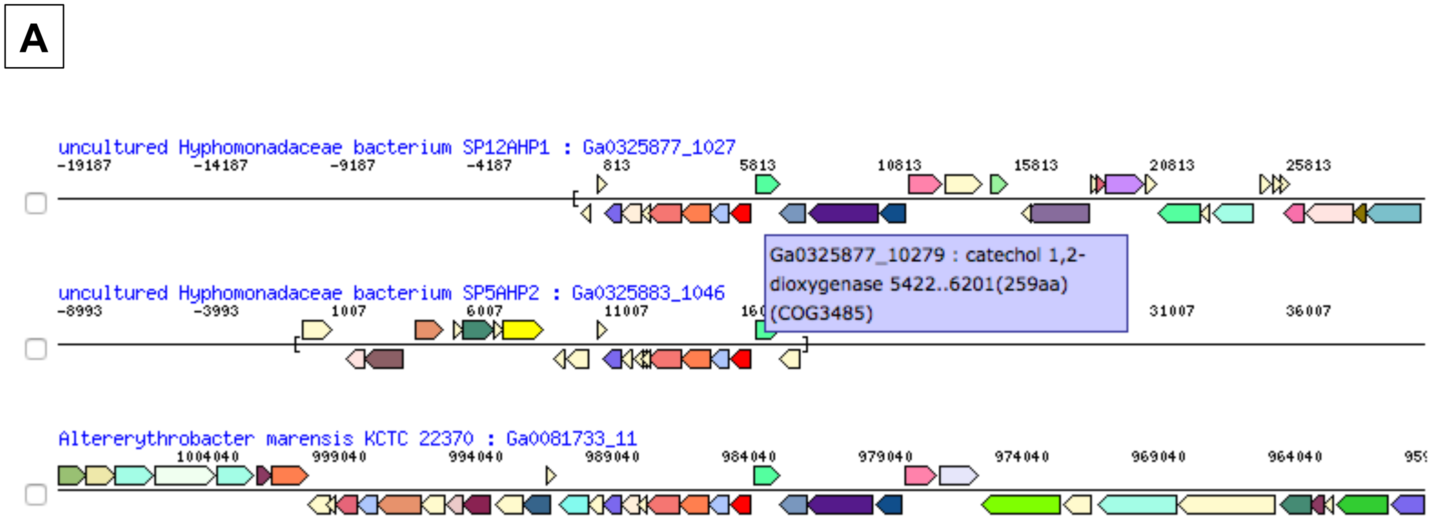

B

locus_tag	start	end	str	aa len	product
Ga0325877_10273	827	1456	-	210	3-oxoacid CoA-transferase subunit B
Ga0325877_10274	1456	2166	-	237	3-oxoacid CoA-transferase subunit A
Ga0325877_10275	2163	2432	-	90	hypothetical protein
Ga0325877_10276	2426	3637	-	404	acetyl-CoA acyltransferase
Ga0325877_10277	3637	4698	-	354	maleylacetate reductase (EC:1.3.1.32)
Ga0325877_10278	4695	5396	-	234	carboxymethylenebutenolidase (EC:3.1.1.45)
Ga0325877_10279	5422	6201	-	260	chlorocatechol 1,2-dioxygenase (EC:1.13.11)
Ga0325877_102710	6347	7234	+	296	AraC-like DNA-binding protein
Ga0325877_102711	7231	8196	-	322	vanillate O-demethylase ferredoxin subunit (EC:1.14.13)
Ga0325877_102712	8287	10815	-	843	iron complex outermembrane receptor protein
Ga0325877_102713	10945	11847	-	301	DNA-binding transcriptional LysR family regulator
Ga0325877_102714	11989	13176	+	396	muconate cycloisomerase (EC:5.5.1.11)
Ga0325877_102715	13280	14659	+	460	Rieske-like 2Fe-2S protein
Ga0325877_102716	15002	15586	+	195	Cu-Zn family superoxide dismutase

C

locus_tag	start	end	str	aa len	product
Ga0325883_104610	9624	10412	-	263	AAA domain-containing protein
Ga0325883_104611	10750	10974	+	75	hypothetical protein
Ga0325883_104612	10988	11617	-	210	3-oxoacid CoA-transferase subunit B
Ga0325883_104613	11617	12072	-	152	3-oxoacid CoA-transferase A subunit
Ga0325883_104614	12084	12245	-	54	coenzyme A transferase
Ga0325883_104615	12084	12360	-	92	hypothetical protein
Ga0325883_104616	12084	12626	-	181	hypothetical protein
Ga0325883_104617	12084	13831	-	582	acetyl-CoA acyltransferase
Ga0325883_104618	12084	14892	-	936	maleylacetate reductase (EC:1.3.1.32)
Ga0325883_104619	12084	15590	-	1169	carboxymethylenebutenolidase (EC:3.1.1.45)
Ga0325883_104620	12084	16395	-	1437	chlorocatechol 1,2-dioxygenase (EC:1.13.11)
Ga0325883_104621	12084	17428	+	1781	AraC-like DNA-binding protein
Ga0325883_104622	12084	18144	-	2020	vanillate O-demethylase ferredoxin subunit

Ga0325883_103332	28948	29029	+	27	tRNA
Ga0325883_103333	29544	30128	-	195	Cu-Zn family superoxide dismutase
Ga0325883_103334	30471	31850	-	460	Rieske-like 2Fe-2S protein
Ga0325883_103335	31954	33141	-	396	muconate cycloisomerase
Ga0325883_103336	33283	34185	+	301	DNA-binding transcriptional LysR family regulator
Ga0325883_103337	34315	36207	+	631	iron complex outermembrane receptor protein

D

locus_tag	start	end	str	aa len	product
Ga0081733_11974	990774	990863	-	-	tRNA
Ga0081733_11973	989592	990704	+	371	ribonuclease Z
Ga0081733_11972	989065	989595	+	177	AhpC/TSA family protein
Ga0081733_11971	988430	989065	+	212	3-oxoadipate CoA-transferase, beta subunit
Ga0081733_11970	987729	988433	+	235	3-oxoacid CoA-transferase, alpha subunit
Ga0081733_11969	987475	987732	+	86	hypothetical protein
Ga0081733_11968	986258	987475	+	406	acetyl-CoA C-acetyltransferase
Ga0081733_11967	985194	986261	+	356	Alcohol dehydrogenase, class IV
Ga0081733_11966	984499	985197	+	233	carboxymethylenebutenolidase
Ga0081733_11965	983647	984429	+	261	chlorocatechol 1,2-dioxygenase
Ga0081733_11964	982628	983536	-	303	AraC-type DNA-binding protein
Ga0081733_11963	981635	982597	+	321	vanillate O-demethylase ferredoxin subunit
Ga0081733_11962	979176	981575	+	800	iron complex outermembrane recepter protein
Ga0081733_11961	978159	979067	+	303	DNA-binding transcriptional LysR family regulator
Ga0081733_11960	976848	978008	-	387	muconate cycloisomerase
Ga0081733_11959	975362	976741	-	460	Phenylpropionate dioxygenase
Ga0081733_11958	972321	975227	+	969	outer membrane autotransporter
Ga0081733_11957	971208	972179	+	324	Helix-turn-helix domain-containing protein
Ga0081733_11956	968082	970967	+	962	zinc protease
Ga0081733_11955	964450	967980	+	1177	TonB-dependent receptor
Ga0081733_11954	963170	964294	+	375	transmembrane sensor
Ga0081733_11953	962644	963183	+	180	RNA polymerase sigma-70 factor
Ga0081733_11952	962311	962469	+	53	hypothetical protein
Ga0081733_11951	960329	962191	+	621	monovalent cation:H+ antiporter
Ga0081733_11950	958779	960191	+	471	dihydrolipoamide dehydrogenase
Ga0081733_11949	958294	958683	+	130	acyl-CoA thioesterase YciA
Ga0081733_11948	957889	958290	+	134	hypothetical protein
Ga0081733_11947	956595	957887	+	431	pyruvate dehydrogenase

Supplementary Figure 12. Phylogenetic tree of halocatechol 1,2 dioxygenases. Tree shows SP12AHP1 and SP5AHP1 halocatechol 1,2-dioxygenases, highlighted in red, compared to closest GenBank nr database protein relatives. Chemically characterized chlorocatechol 1,2-dioxygenases are highlighted in blue.

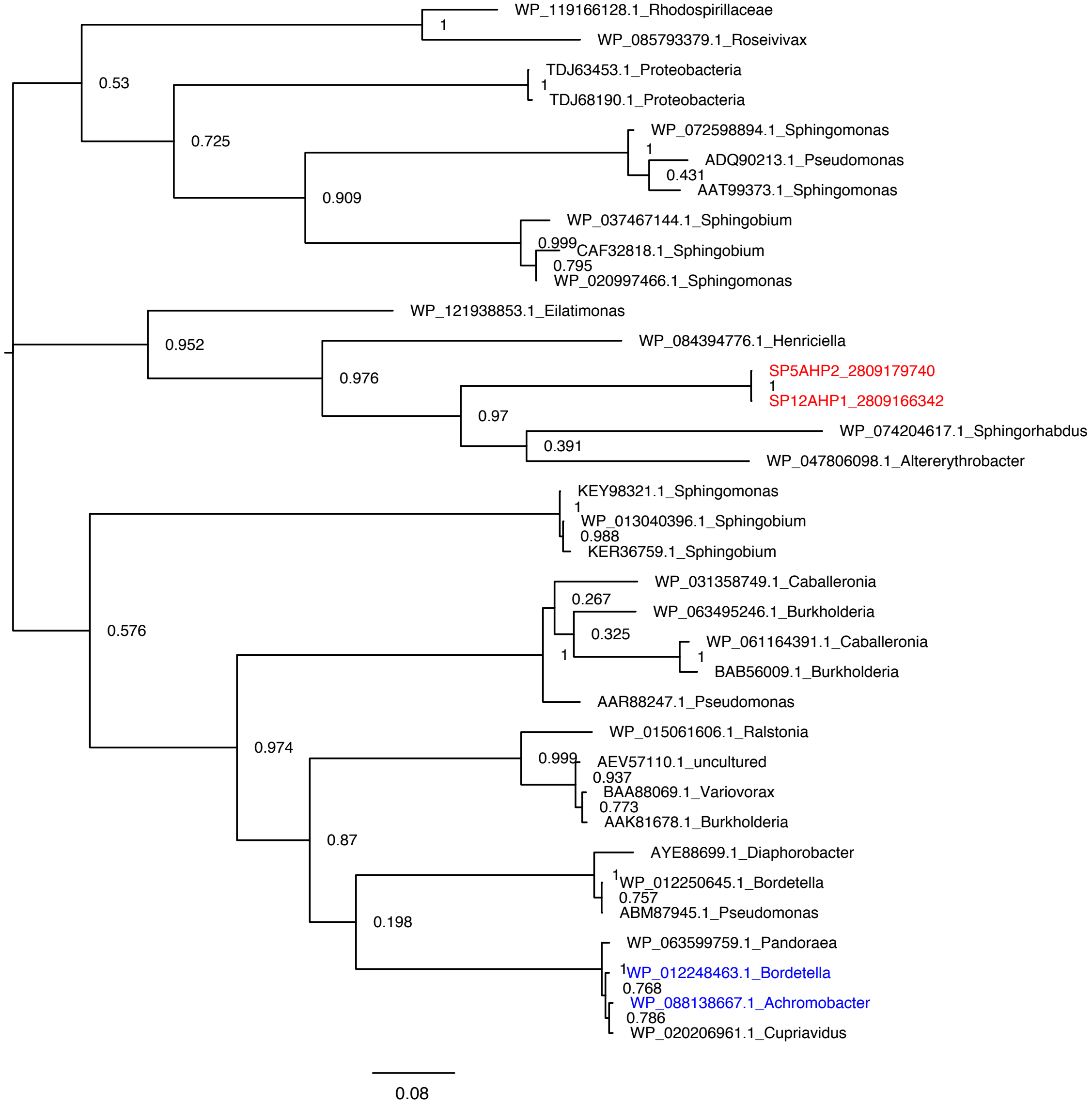

Supplementary Figure 13. MySQL schema for extracting sample metadata and abundance statistics from the Sponge Microbiome Database. Tables were populated using data in Supplementary tables 3,4, and 5 from [10].

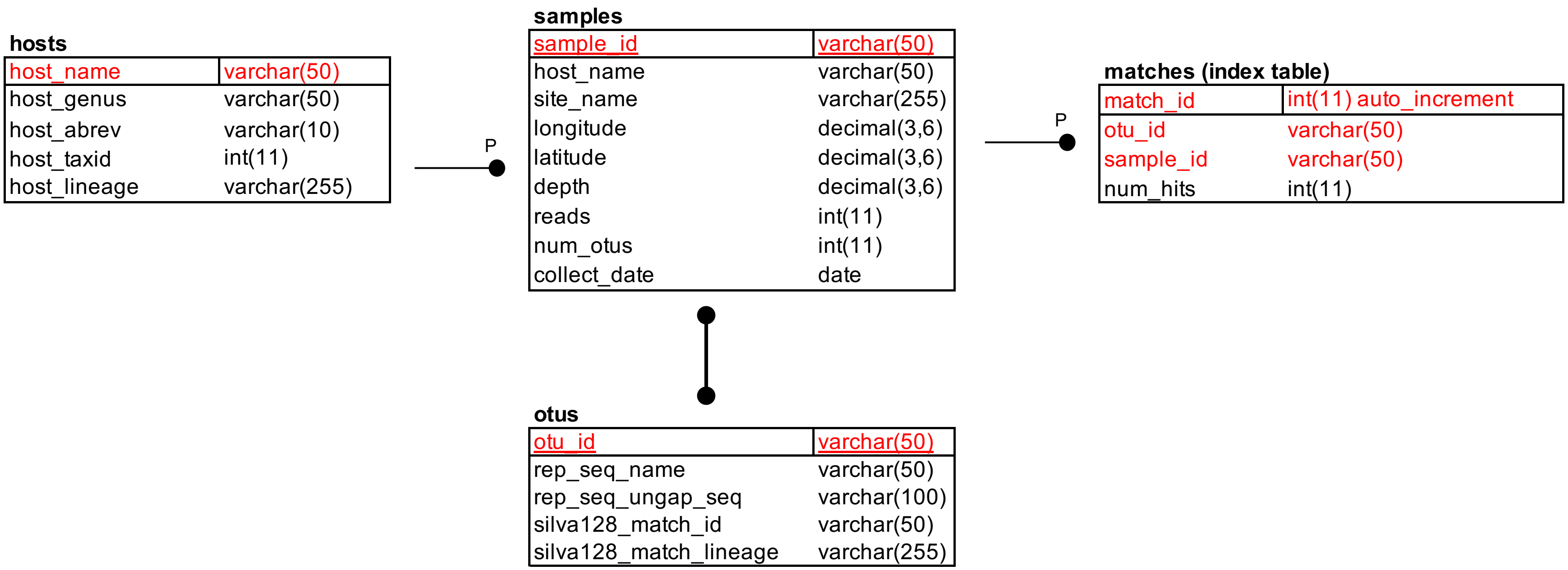

Supplementary Table 1. *Lamellodysidea* sponge sampling metadata.

Host clade (ITS)	Sample name	Location	Collect date	Description	NCBI Biosample ID	16S amplicon merged seqs	Illumina metagenomic reads	PacBio metagenomic reads
Ia	SP12	Anae Island	June 2014	lime green, purple patches, flat	SAMN04939363	227,847	45,810,804	-
Ia	SP5	Piti Bomb Holes	June 2014	lime green, purple base, lettuce leaf	SAMN04939377	126,418	46,496,996	-
Ia	SP1	Piti Bomb Holes	June 2014	lime green, purple base, lettuce leaf	SAMN04939374	97,761	-	-
Ia	SP8	Piti Bomb Holes	June 2014	lime green, purple base, lettuce leaf	SAMN04939378	112,399	-	-
Ia	GUM038	Anae Island	July 2015	white/gray, flat	SAMN04939360	114,867	-	-
Ia	GUM040	Anae Island	July 2015	white/gray, flat	SAMN04939361	213,095	-	-
Ia	GUM058	Piti Bomb Holes	July 2015	lime green, lettuce leaf	SAMN04939368	84,608	-	-
Ia	GUM096	Piti Bomb Holes	July 2015	lime green, lettuce leaf	SAMN04939369	114,282	-	-
Ia	GUM203	Piti Bomb Holes	Dec. 2016	lime green, purple base, lettuce leaf	SAMN12982864	3,280	-	-
Ib	GUM020	Piti Bomb Holes	July 2015	green with white ridged fingers	SAMN04939367	123,293	-	-
Ib	GUM102	Piti Bomb Holes	July 2015	green with white ridged fingers	SAMN04939373	195,184	148,726,466	-
Ib	GUM204	Tumon Bay	Dec. 2016	green with white ridged fingers	SAMN12982865	6,503	-	-
II	GUM201	Anae Island	Dec. 2016	green flat with delicate fingers	SAMN12982858	5,096	-	-
II	GUM202	Anae Island	Dec. 2016	white/gray flat with delicate fingers	SAMN10266269	4,237	100,368,352	31,034
III	GUM069	Pago Bay	July 2015	dark green ridged, fleshy fingers	SAMN04939366	128,996	-	-
III	GUM7D2	Pago Bay	July 2015	dark green ridged, fleshy fingers	SAMN04939364	79,191	178,947,320	-
III	GUM007	Pago Bay	July 2015	trichome enrichment of sample GUM7D2	SAMN04939365	-	82,554,626	180,838
na	GUM61	Anae Island	July 2015	seawater	SAMN12983225	1,405,063	-	-

Supplementary Table 2. Sponge holobiont and trichome metagenomic assembly statistics.

Sample name	Sample type	pct reads assembled	Read type	Avg read length	Assembled reads	scf >3K	Max scf len
SP12	holobiont tissue	32%	Illumina	90	14,534,232	9,939	75,936
SP5	holobiont tissue	37%	Illumina	90	17,349,474	5,437	235,084
GUM102	holobiont tissue	46%	Illumina	135	68,102,612	16,294	75,068
GUM202	holobiont tissue	75%	Illumina	150	75,290,051	34,352	442,614
GUM7D2	holobiont tissue	33%	Illumina	150	27,376,842	30,974	325,242
GUM202	trichome enrichment	nd	PacBio	3852	31,034	67*	304,029*
GUM007	trichome enrichment	40%	PacBio	4445	71,965	6	3,147,220

*GUM202 trichome enrichment reads were assembled in combination with Illumina GUM202 holobiont sequences, as previously described in [11].

Supplementary Table 3. MIMAG parameters for assembled genomes.

Host clade	Assembly Name	Organism name	analysis project type	assemb. software	Multi-marker taxonomy	MIMAG qual	Completeness score	Contamination score	Completeness software
Ia	SP12AHP1	Hyphomonadaceae bacterium SP12AHP1	MAG	idba-ud v. 1.1.1	Alphaproteobacteria;Rhodobacterales; Hyphomonadaceae	high	91.4	0.0	Check M
Ia	SP12ARB1	Rhodobacteraceae bacterium SP12ARB1	MAG	idba-ud v. 1.1.1	Alphaproteobacteria;Rhodobacterales; Rhodobacteraceae	med	69.1	0.0	Check M
Ia	SP12BCY1	Ekhidna sp. SP12BCY1	MAG	idba-ud v. 1.1.1	Bacteroidetes;Cytophagia;Cytophagales; Cytophagaceae;Ekhidna	high	100.0	0.0	Check M
Ia	SP12CHS1	Hormopscilla sp. SP12CHS1	MAG	idba-ud v. 1.1.1	Cyanobacteria;Oscillatoriophycideae; Oscillatoriales;Gomontiellaceae;Hormoscilla	high	96.6	0.2	Check M
Ia	SP5AHP2	Hyphomonadaceae bacterium SP5AHP1	MAG	Celera Assem. v.8.3	Alphaproteobacteria;Rhodobacterales; Hyphomonadaceae	med	87.8	0.0	Check M
Ia	SP5ARB1	Rhodobacteraceae bacterium SP5ARB1	MAG	idba-ud v. 1.1.1	Alphaproteobacteria;Rhodobacterales; Rhodobacteraceae	med	82.4	3.4	Check M
Ia	SP5ARS3	Rhodospirllales bacterium SP5ARS3	MAG	idba-ud v. 1.1.1	Alphaproteobacteria:Rhodospirillales	med	94.8	0.0	Check M
Ia	SP5BCY1	Ekhidna sp. SP5BCY1	MAG	idba-ud v. 1.1.1	Bacteroidetes;Cytophagia;Cytophagales; Cytophagaceae;Ekhidna	high	96.4	0.5	Check M
Ia	SP5CHS1	Hormopscilla sp. SP5CHS1	MAG	idba-ud v. 1.1.1	Cyanobacteria;Oscillatoriophycideae; Oscillatoriales;Gomontiellaceae;Hormoscilla	high	96.6	0.2	Check M
Ia	SP5CPC1	Pochlorn sp SP5CPC1	MAG	idba-ud v. 1.1.1	Cyanobacteria;Synechococcales;Prochloraceae; Prochloron	med	77.4	0.0	Check M
Ia	SP5GCR1	Chromatiales bacterium SP5GCR1	MAG	idba-ud v. 1.1.1	Gammaproteobacteria;Chromatiales	high	98.3	0.0	Check M
Ia	SP5OBV1	Bdellovibrionales bacterium SP5)BV1	MAG	idba-ud v. 1.1.1	Oligoflexia;Bdellovibrionales	high	91.2	0.0	Check M
Ib	GM102ARS1	Rhodospirillales bacterium GM102ARS1	MAG	idba-ud v. 1.1.1	Alphaproteobacteria:Rhodospirillales	low	41.2	0.8	Check M
Ib	GM102HS1	Hormopscilla sp. GM102CHS1	MAG	idba-ud v. 1.1.1	Cyanobacteria;Oscillatoriophycideae; Oscillatoriales;Gomontiellaceae;Hormoscilla	med	95.7	1.8	Check M
II	GM202ARS1	Rhodospirillales bacterium GM202ARS1	MAG	Celera Assem.	Alphaproteobacteria:Rhodospirillales	high	100.0	0.0	Check M

Host clade	Assembly Name	Organism name	analysis project type	assemb. software	Multi-marker taxonomy	MIMAG qual	Completeness score	Contamination score	Completeness software
				v.8.3					
II	GM202ARS2	Candidatus Methylospongiales bacterium GM202ARS2	MAG	idba-ud v. 1.1.1	Alphaproteobacteria:Candidatus Methylospongiales	med	85.3	0.8	CheckM
II	GM202BCY1	Ekhidna sp. GM202BCY1	MAG	Celera Assem. v.8.3	Bacteroidetes;Cytophagia;Cytophagales; Cytophagaceae;Ekhidna	high	94.9	0.5	CheckM
II	GM202CHS1	Hormoscilla sp. GM202CHS1	MAG	SPAdes v. 3.10.1	Cyanobacteria;Oscillatoriophycideae; Oscillatoriales;Gomontiellaceae;Hormoscilla	high	97.4	2.0	CheckM
III	GM7ARB1	Rhodobacteraceae bacterium GM7ARB1	MAG	Celera Assem. v.8.3	Alphaproteobacteria;Rhodobacterales; Rhodobacteraceae	high	96.6	0.0	CheckM
III	GM7ARB2	Rhodobacteraceae bacterium GM7ARB2	MAG	Celera Assem. v.8.3	Alphaproteobacteria;Rhodobacterales; Rhodobacteraceae	high	93.7	0.9	CheckM
III	GM7ARS4	Candidatus Methylospongiales bacterium GM7ARS2	MAG	idba-ud v. 1.1.1	Alphaproteobacteria:Candidatus Methylospongiales	med	91.0	0.8	CheckM
III	GM7CHS1pb	Hormopscilla sp. GM7CHS1	MAG	CANU v. 1.8	Cyanobacteria;Oscillatoriophycideae; Oscillatoriales;Gomontiellaceae;Hormoscilla	high	100.0	1.8	CheckM
III	GM7GCV1	Cellvibrionales bacterium GM7GCV1	MAG	Celera Assem. v.8.3	Gammaproteobacteria;Cellvibrionales	med	89.1	0.0	CheckM

Supplementary Table 4. Closest environmental matches from the GenBank nr database to 16S rRNA genes obtained from MAGs. GM102CHS1, GM7ARS4, SP5ARS3, GM102ARS1 are not included in this table because they did not include any 16S rRNA genes of 300 nt or longer.

Tax group	MAG	Environ 16S tophit	Env. 16S pct ident	Aln len	Environ tophit habitat	Ref.	Lineage
CHS	GM202CHS1	AY615507.1	99%	1410	Sponge - Lamellodysidea	[12]	Cyanobacteria;Oscillatoriophycideae;Oscillatoriales; Gomontiellaceae;Hormoscilla
CHS	GM7CHS1	AY615501.1	100%	1410	Sponge - Lamellodysidea	[12]	Cyanobacteria;Oscillatoriophycideae;Oscillatoriales; Gomontiellaceae;Hormoscilla
CHS	SP12CHS1	AY615505.1	99%	1410	Sponge - Lamellodysidea	[12]	Cyanobacteria;Oscillatoriophycideae;Oscillatoriales; Gomontiellaceae;Hormoscilla
CHS	SP5CHS1	AY615505.1	100%	1410	Sponge - Lamellodysidea	[12]	Cyanobacteria;Oscillatoriophycideae;Oscillatoriales; Gomontiellaceae;Hormoscillaa
CPC	SP5CPC1	DQ357958.1	99%	1443	Tunicates - Lissoclinum	[13]	Cyanobacteria;Synechococcales;Prochloraceae;Prochloron
AMS	GM202ARS2	AY845234.1	99%	1425	Sponge - Lamellodysidea	[12]	Alphaproteobacteria:Candidatus Methylospongiales
AMS	GM202ARS1	AY845233.1	100%	1468	Sponge - Lamellodysidea	[12]	Alphaproteobacteria:Rhodospirillales
ARB	SP5ARB1	AY845235.1	99%	1384	Sponge - Lamellodysidea	[12]	Alphaproteobacteria;Rhodobacterales;Rhodobacteraceae
ARB	SP12ARB2	AY845235.1	98%	1379	Sponge - Lamellodysidea	[12]	Alphaproteobacteria;Rhodobacterales;Rhodobacteraceae
ARB	GM7ARB1	KM051808.1	97%	1448	Basaltic Crust Borehole biofilm	Un-published	Alphaproteobacteria;Rhodobacterales;Rhodobacteraceae
ARB	GM7ARB2	CP016093.1	88%	1474	Biocathode biofilm	Un-published	Alphaproteobacteria;Rhodobacterales;Rhodobacteraceae
AHP	SP12AHP1	JF769570.1	96%	1467	seawater near algae (sea lettuce)	Un-published	Alphaproteobacteria;Rhodobacterales;Hyphomonadaceae
AHP	SP5AHP2	JF769570.1	96%	1467	seawater near algae (sea lettuce)	Un-published	Alphaproteobacteria;Rhodobacterales;Hyphomonadaceae
BCY	GM202BCY1	AM259890.1	96%	1460	sponge - Tethya	[14]	Bacteroidetes;Cytophagia;Cytophagales;Cytophagaceae
BCY	SP5BCY1	AM259890.1	96%	1460	sponge - Tethya	[14]	Bacteroidetes;Cytophagia;Cytophagales;Cytophagaceae
BCY	SP12BCY1	AM259890.1	96%	1460	sponge - Tethya	[14]	Bacteroidetes;Cytophagia;Cytophagales;Cytophagaceae
GCR	SP5GCR1	JX391667.1	91%	1514	PBDE contam marine sediment	Un-published	Gammaproteobacteria;Chromatiales
GCV	GM7GCV1	JQ062844.1	90%	1500	sponge- Stylissa	[15]	Gammaproteobacteria;Cellvibrionales;Cellvibrionaceae
OVB	SP5OBV1	EF092260	96%	1442	sponge - Axinella	Un-published	Oligoflexia;Bdellovibrionales

Supplementary Table 5. Assembled MAG 16S rRNA gene matches to Sponge Microbiome Database samples at 97% nucleotide identity. Database sequences were 99 bp segments amplified from the V4 region of the 16S rRNA gene [10]. Sponge Microbiome Database samples with the maximum number of matches to each MAG-associated 16S rRNA gene from this study were selected to determine the number and percent of matched sequences, and reported here along with host genus and geographical collection site. GM102CHS1, GM7ARS4, SP5ARS3, GM102ARS1 are not included in this table because they did not include any sequences overlapping the V4 region of the 16S rRNA gene.

Tax group	MAGs	16S len	All host genera	Host with max pct	total OTU in sample	max num OTU matches	max pct OTU matches	site name	sample_id
CHS	SP12CHS1, SP5CHS1, GM202CHS1, GM7CHS1	1486	Xestospongia, Cliona	Xestospongia bocatorensis	647	57	16.7%	Panama	P10X53.1020516
BCY	GUM202BCY1, SP5BCY1, SP12BCY1	1524	Carteriospongia, Cymbastela, Dysidea, Ectyoplasia, Mycale, marine sediment	Cymbastela coralliophila	304	4	1.3%	Davies Reef	Webster.1.E1.49.1019860
ARB	GM7ARB2	1243	Dysidea	Dysidea avara	336	15	4.5%	Limski Canal	GC.4.2.1019709
ARB	GM7ARB1	1470	Chondrilla, Cinachyrella, Cliona, Dysidea, Ectyoplasia, Lissodendoryx, Mycale, seawater, marine sediment	Lissodendoryx colombiensis	237	1	0.4%	Panama	P12x59.1019532
ARB	SP5ARB1, SP12ARB1	1497	Dysidea, Xestospongia	Xestospongia bocatorensis	678	1	0.1%	Panama	SI06.11.1019752
ARS	GM202ARS2	1504	Dysidea	Dysidea avara	336	2	0.6%	Limski Canal	GC.4.2.1019709
ARS	GUM202ARS1	1507	Cymbastela. Xestospongia	Cymbastela coralliophila	1341	3	0.2%	Davies Reef	SS.9.2.1019758
AHP	SP12AHP1 SP5AHP2	1479	Ircinia	Ircinia strobilina	2940	1	0.04%	Banco Chinchorros	17.1020038
DBV	SP5DBV1	1517	Cymbastela, Ircinia, Mycale	Cymbastela	1085	3	0.3%	Sponge Farm	ZA.44.1020227
GCR	SP5GCR1	1548	Carteriospongia, Cymbastela, Cliona, Xestospongia	Carteriospongia foliascens	953	4	0.4%	Kimberley, Western Australia	Webster.3.H4.276.1019944

Tax group	MAGs	16S len	All host genera	Host with max pct	total OTU in sample	max num OTU matches	max pct OTU matches	site name	sample_id
GCV	GM7GCV1	1517	no matches above threshold	nd	nd	nd	nd	nd	nd
CPC	SP5CPC1	1442	no matches above threshold	nd	nd	nd	nd	nd	nd

Supplementary Table 6. Tally of membrane-associated gene products in MAGs. Values for adhesive genes, signal sequences, and transporter types were tallied based on keyword searches of MAG annotations at IMG-MER [7].

tax group	Genome name	num CDS	num trans-porters	pct CDS as trans-porter	chemo-taxis proteins (Che/Mot)	motility type	cadherin domain proteins	ankyrin domain proteins	Type IV pili genes	num signal peptide genes	pct signal peptide genes	signal proteases	signal glycosidases
CHS	SP12CHS1	5961	195	3.3%	6	twitching		2	12	74	1.2	5	0
CHS	SP5CHS1	5738	176	3.1%	4	twitching		2	11	80	1.4	7	0
CHS	GM102CHS1	6390	172	2.7%	2	twitching	1	2	16	78	1.2	6	0
CHS	GM7CHS1	7441	196	2.6%	5	twitching	2	3	13	111	1.4	6	1
CHS	GM202CHS1	6160	247	4.0%	6	twitching		5	15	113	1.5	7	0
CPC	SP5CPC1	3413	122	3.6%	24	twitching		5	8	83	2.4	4	1
AMS	GM7ARS4	1377	76	5.5%	0	none		3	8	57	4.0	4	0
AMS	GM202ARS2	1836	91	5.0%	0	none		18	11	88	4.7	6	0
ARS	GM202ARS1	2858	251	8.8%	1	none		14	2	136	4.7	5	2
ARS	SP5ARS3	2570	312	12.1%	0	none		22		146	5.6	2	2
ARS	GM102ARS1	1161	102	8.8%	1	none		8		47	4.0	2	1
ARB	GM7ARB1	2449	247	10.1%	2	none		14		124	5.0	4	0
ARB	GM7ARB2	2505	234	9.3%	1	none		8	1	129	5.1	5	3
ARB	SP12ARB1	3273	263	8.0%	1	none	1	6	2	121	3.7	5	1
ARB	SP5ARB1	2628	237	9.0%	1	none		6	2	130	4.9	1	1
AHP	SP12AHP1	2275	113	5.0%	0	none		30		152	6.5	7	7
AHP	SP5AHP2	2292	107	4.7%	0	none		32		135	5.8	6	6
BCY	SP12BCY1	2828	128	4.5%	12	gliding	29	10	1	147	5.1	12	4
BCY	SP5BCY1	2774	126	4.5%	12	gliding	31	8	1	142	5.1	9	4
BCY	GM202BCY1	2879	125	4.3%	12	gliding	30	11	1	134	4.6	9	3
GCR	SP5GCR1	1474	91	6.2%	0	none	13		8	70	4.6	4	2
GCV	GM7GCV1	1744	109	6.3%	1	twitching		2	10	123	6.9	5	3
OVB	SP5OBV1	1744	66	3.8%	0	twitching		31	13	60	3.9	1	0

Supplementary Table 7. Viral defense genes in assembled MAGs. Values were tallied based on keyword searches of MAG annotations at IMG-MER [7].

tax group	MAG	CRISPR loci (Minced)	integrated prophage recovered	IMG trans-posases	IMG restriction endo-nuclease	IMG DNA-modiification methylase	toxin/anti-toxin genes
CHS	SP12CHS1	5	0	60	7	6	81
CHS	SP5CHS1	14	0	50	51	16	78
CHS	GM102CHS1	8	2	117	67	20	67
CHS	GM202CHS1	4	1	318	51	11	93
CHS	GM7CHS1	5	2	226	113	6	91
CPC	SP5CPC1	0	0	73	12	8	29
AMS	GM202ARS2	5	3	38	6	7	10
AMS	GM7ARS4	6	0	1	4	5	1
ARS	GM202ARS1	11	0	4	6	3	3
ARS	SP5ARS3	14	0	1	7	3	2
ARS	GM102ARS1	10	1	0	1	1	1
ARB	SP5ARB1	6	0	15	9	0	0
ARB	SP12ARB1	12	0	11	4	15	3
ARB	GM7ARB1	16	0	0	5	21	7
ARB	GM7ARB2	27	0	7	5	23	4
AHP	SP12AHP1	15	0	34	7	7	6
AHP	SP5AHP2	13	0	66	117	8	7
BCY	GM202BCY1	8	0	33	9	24	14
BCY	SP5BCY1	5	0	20	9	27	16
BCY	SP12BCY1	9	0	19	7	30	16
GCR	SP5GCR1	11	0	9	3	7	2
GCV	GM7GCV1	14	1	6	6	21	6
OVB	SP5OBV1	24	0	28	4	14	1

Supplementary Table 8. Biosynthetic gene clusters predicted by AntiSmash [16], supplemented with aromatic halogenases predicted by Pfam database model PF04820 [17].

tax group	genome	Type I PKS	NRPS	Type III PKS	terpene-phosphonate	terpene carotenoid	terpene other	bacter-iocin (RiPP)	beta-lactone	PDBE	Aromatic halogenases (PF04820)	cyano-bactin (RiPP)	Lanthi-peptide	Lasso-peptide (RiPP)
CHS	SP12CHS1	1	1				1	1		1		1		
CHS	SP5CHS1		2					1		1		1		
CHS	GM102CHS1	1					1	1		1				
CHS	GM7CHS1pb	3	5				2	1			1	1	1	
CHS	GM202CHS1	4	7				2	1		1	1	1		
CPC	SP5CPC1		2				4	2			1			1
AMS	GM202ARS2				1		1							
AMS	GM7ARS4				1									
ARS	SP5ARS3						1	1						
ARS	GM202ARS1						1							
ARS	GM102ARS1						1	1						
ARB	GM7ARB1					1	1	1						
ARB	GM7ARB2					1		1						
ARB	SP12ARB1					1	1	1			1			
ARB	SP5ARB1					1	1	1			1			
AHP	SP5AHP2					1	1				11			
AHP	SP12AHP					1	1				8			
BCY	SP12BCY1			1		1	1							
BCY	GM202BCY1			1		1	1							
BCY	SP5BCY1			1		1	1							
GCR	SP5GCR1							2						
GCV	GM7GCV1							1	1					
OVB	SP5OVB1													

References

1. Segata, N., et al., *PhyloPhlAn is a new method for improved phylogenetic and taxonomic placement of microbes.* Nat Commun, 2013. **4**: p. 2304.
2. Rodriguez-R, L. and K. Konstantinidis, *The enveomics collection: a toolbox for specialized analyses of microbial genomes and metagenomes.* PeerJ Preprints, 2016. **4**: p. e1900v1.
3. Rodriguez-R, L.M., and Konstantinos T. Konstantinidis, *Bypassing Cultivation To Identify Bacterial Species.* Microbe, 2014. **9**(3): p. 111-8.
4. Yarza, P., et al., *Uniting the classification of cultured and uncultured bacteria and archaea using 16S rRNA gene sequences.* Nat Rev Microbiol, 2014. **12**(9): p. 635-45.
5. Nazir, R., et al., *The Ecological Role of Type Three Secretion Systems in the Interaction of Bacteria with Fungi in Soil and Related Habitats Is Diverse and Context-Dependent.* Front Microbiol, 2017. **8**: p. 38.
6. Dewoody, R.S., P.M. Merritt, and M.M. Marketon, *Regulation of the Yersinia type III secretion system: traffic control.* Front Cell Infect Microbiol, 2013. **3**: p. 4.
7. Chen, I.A., et al., *IMG/M: integrated genome and metagenome comparative data analysis system.* Nucleic Acids Res, 2017. **45**(D1): p. D507-D516.
8. Neubauer, P.R., et al., *A flavin-dependent halogenase from metagenomic analysis prefers bromination over chlorination.* PLoS One, 2018. **13**(5): p. e0196797.
9. Agarwal, V., et al., *Biosynthesis of polybrominated aromatic organic compounds by marine bacteria.* Nat Chem Biol, 2014. **10**(8): p. 640-7.
10. Thomas, T., et al., *Diversity, structure and convergent evolution of the global sponge microbiome.* Nat Commun, 2016. **7**: p. 11870.
11. Schorn, M.A., et al., *Comparative Genomics of Cyanobacterial Symbionts Reveals Distinct, Specialized Metabolism in Tropical Dysideidae Sponges.* MBio, 2019. **10**(3): p. e00821-19.
12. Ridley, C.P., D. John Faulkner, and M.G. Haygood, *Investigation of Oscillatoria spongeliae-dominated bacterial communities in four dictyoceratid sponges.* Appl Environ Microbiol, 2005. **71**(11): p. 7366-75.
13. Donia, M.S., et al., *Complex microbiome underlying secondary and primary metabolism in the tunicate-Prochloron symbiosis.* Proc Natl Acad Sci U S A, 2011. **108**(51): p. E1423-32.
14. Thiel, V., et al., *Spatial distribution of sponge-associated bacteria in the Mediterranean sponge Tethya aurantium.* FEMS Microbiol Ecol, 2007. **59**(1): p. 47-63.
15. Giles, E.C., et al., *Bacterial community profiles in low microbial abundance sponges.* FEMS Microbiol Ecol, 2013. **83**(1): p. 232-41.
16. Blin, K., et al., *antiSMASH 5.0: updates to the secondary metabolite genome mining pipeline.* Nucleic Acids Res, 2019. **47**(W1): p. W81-W87.
17. Finn, R.D., et al., *The Pfam protein families database: towards a more sustainable future.* Nucleic Acids Res, 2016. **44**(D1): p. D279-85.
